# Utilized Distributed Optical Fiber Sensor with Spiral‐Serpentine Deployment Enabling High‐Precision Full‐Field Temperature Reconstruction and Thermal Management for Pouch Lithium‐Ion Battery

**DOI:** 10.1002/advs.202511030

**Published:** 2025-09-04

**Authors:** Yuhao Zhu, Xiaoqiang Zhang, Yunlong Shang, Miao Yu, Xin Gu, Jinglun Li, Linfei Hou

**Affiliations:** ^1^ School of Control Science and Engineering Shandong University Jinan 250061 China

**Keywords:** distributed optical fiber sensor, electric vehicles, lithium‐ion battery, temperature distribution and reconstruction, thermal management, uncertainty quantification

## Abstract

Real‐time and accurate temperature monitoring has been widely recognized in both academia and industry to ensure battery operation safety. Traditional techniques are generally limited to incomplete information caused by discrete sampling points. Hence, the spiral‐serpentine distributed optical fiber sensor (DOFS) layout is presented to realize in‐situ full‐range temperature measurement. Unlike conventional contact‐based sensors, DOFS offers high spatial resolution with 1.28 mm for comprehensive‐accurate monitoring. The proposed deployment enables mapping across the entire surface, rather than being restricted to certain points or localized regions. Meanwhile, the locally adaptive radial basis function interpolation algorithm is developed to reconstruct temperature filed, which aims to ensure the global smoothness and local variability. Uncertainty quantification is incorporated to enhance the results reliability. Experimental studies are conducted on large‐format pouch LIBs used in BYD electric vehicles under various currents. The results demonstrate that it can accurately and in real‐time capture temperature variations. The developed reconstruction method precisely acquires the full‐field temperature distribution with a max standard deviation below 0.3 ℃. Detailed comparison with other six measurement‐reconstruction methods such as thermocouple (TC), infrared thermography (IT), Fiber Bragg Grating (FBG) and different‐shaped DOFS further highlights the superiority. This work offers significantly valuable insights for optimizing battery thermal management systems.

## Graphical abstract

Real‐time and accurate monitoring of battery surface temperature is widely recognized as essential for ensuring operational safety. By synergistically combining high‐resolution sensing‐interrogation systems, optimized DOFS deployment scheme, and intelligent reconstruction algorithms, this study achieves full‐range and high‐fidelity temperature field reconstruction with an error below 0.3 °C, offering critical guidance for the next‐generation battery design and thermal management systems optimization.

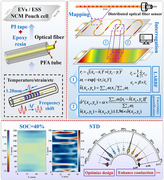

## 1 Introduction

**Figure 1:**
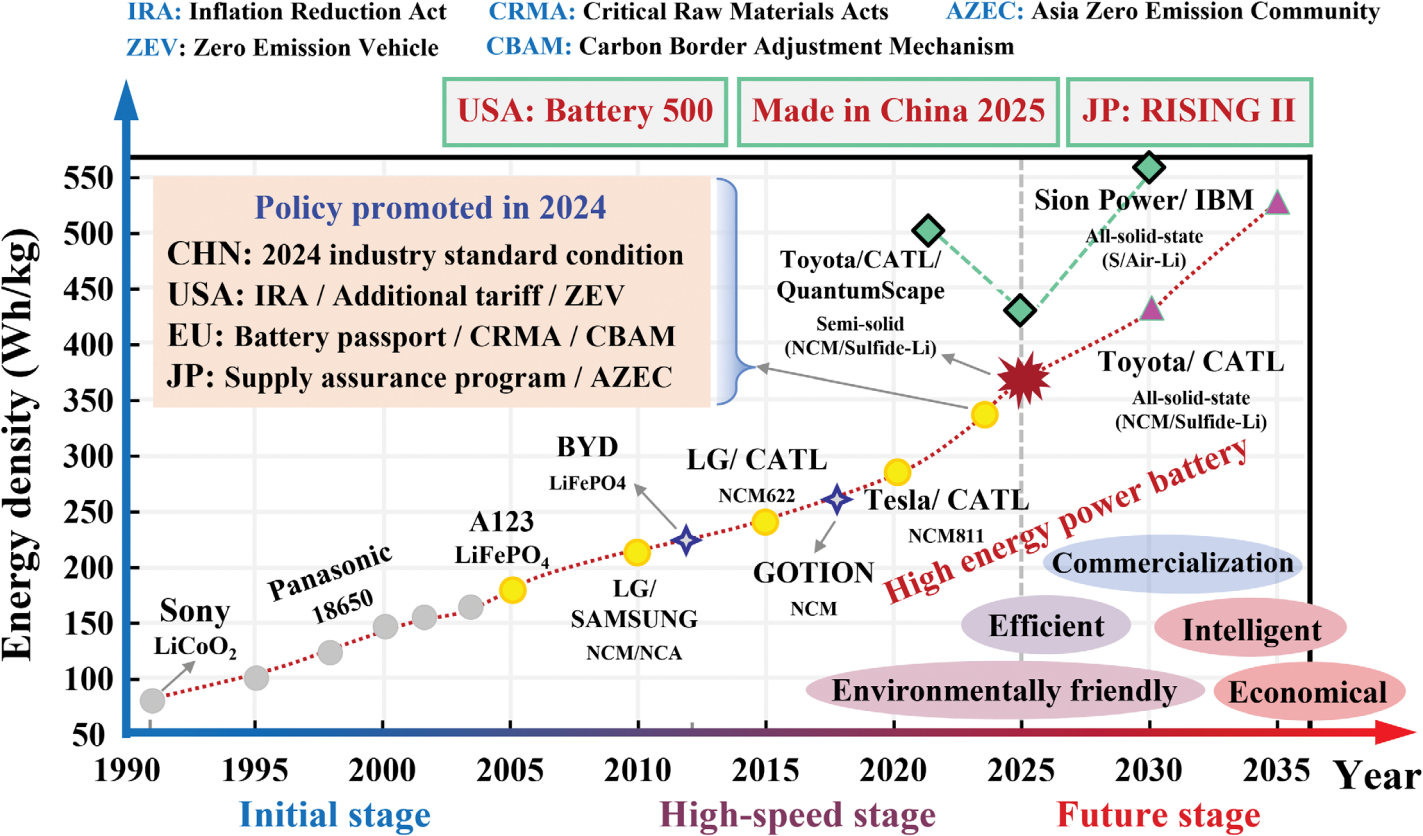
Commercial lithium‐ion battery development roadmap.

Traditional fossil energy resources are gradually depleting and no longer suitable for sustainable development. The energy structure is essential to transform to achieve carbon neutrality. Lithium‐ion battery (LIB), with the high energy density, long cycle life, and zero pollution, are widely regarded as strong candidates to accomplish this goal,^[^ ^1^, ^2^ ^]^ which has significant market potential in electric vehicles (EVs) and renewable energy storage systems (ESS). According to EVTank, the global shipment of LIBs in 2024 reached 1545.1GWh, with production value exceeding 140 billion USD. Meanwhile, the next‐generation battery, with enhanced capacity (>600Ah), higher energy density (>400 Wh/kg), and longer life (>10 000 cycles), is being pursued globally, as shown in **Figure** **1**. Multiple policy frameworks such as 2024 Industry Standard Condition in China, Battery Passport Regulation and the Critical Raw Materials Act in European Union (EU) have injected continuous impetus into this process, which aim to achieve ongoing breakthroughs in energy density while ultimately realizing the vision of environmentally friendly, costeffective, efficient, and intelligent batteries by leveraging policy incentives and industry collaboration as key drivers. However, critical challenges remain in system safety and reliability, necessitating further optimization.^[^ ^3^ ^]^ A primary contributing factor is the insufficient exploration and understanding of the thermal‐mechanical‐electrochemical behaviors and particularly complex coupled relations.

In fact, LIBs always exhibit inherent multi‐physics coupling characteristics which involve electrochemical, thermal, and mechanical fields interactions.^[^ ^4^ ^]^ During charge and discharge processes, Li+ repeatedly intercalate and remove, with internal resistance, polarization, and side reactions dominating heat generation. Simultaneously, the asynchronous volume changes of the cathode and anode, along with the formation of solid electrolyte interface layer, always induce stress and strain, which leads to electrode progressive fatigue, electrolyte decomposition, and gas evolution.^[^ ^5^, ^6^ ^]^ The stress and internal resistance are increased to cause battery expansion. Elevated internal resistance further intensifies heat generation, while material thermal expansion exacerbates strain. Strain can also impair the contact between the electrode and the electrolyte, which causes the resistance further increasing and form vicious cycle that ultimately produces inevitable failure or even severe thermal runaway events.^[^ ^7^ ^]^ Hence, given the prevailing trend toward large‐format battery designs, a comprehensive investigation of thermal behavior is imperative. Advanced characterization of temperature evolution will enable precise regulation strategies to provide support for improving system performance and safe operation.

The sensor technology plays a vital role in monitoring physical–chemical signals, which serves for the health and safety state evaluation of LIBs. Traditional thermal monitoring primarily relies on compact and easily deployable devices such as thermocouple (TC)^[^ ^8^, ^9^, ^10^, ^11^, ^12^, ^13^ ^]^ which are always fixed at predefined positions. However, owing to the factors such as noise generated by electromagnetic interference^[^ ^14^ ^]^ and poor environmental adaptability,^[^ ^15^ ^]^ a series of challenges arise in the installation and maintenance of increasingly complex‐miniaturized systems. Recently, the European Union's “Battery 2030+” initiative highlighted that measurement technique integrated optical fiber sensor (OFS) is essential for the development of future smart battery.^[^ ^16^ ^]^ Compared to traditional sensors, OFS offers some advantages such as higher resolution, better adaptability, and enhanced anti‐interference capabilities, which have gradually been adopted for thermal‐mechanical monitoring.^[^ ^17^ ^]^ Fiber Bragg Grating sensors (FBGs), one of the most extensively studied OFS, operate based on the principle of light selective reflection. The reflected light varies in response to temperature/strain changes. In fact, as early as 2013, Yang et al.^[^ ^18^ ^]^ were the first to integrate FBGs with batteries, which demonstrated that FBG exhibited superior thermal response performance compared to TC, marking a significant milestone in the field of battery measurement. Subsequently, Pinto et al.^[^ ^19^ ^]^ employed four FBGs to monitor external temperature variations under various current rates. The results presented that it has excellent responsiveness to detect the temperature changes. Nascimento et al.^[^ ^20^ ^]^ utilized FBG to track temperature changes at five distinct locations on the surface, which enables the creation of thermal map. Similarly, Peng et al.^[^ ^21^ ^]^ combined the metal ring and FBG sensors to form a new encapsulation structure to monitor the battery thermal behavior, which has excellent repeatability through experimental results. Li et al.^[^ ^22^ ^]^ proposed an intelligent battery integrated with FBG sensor, which enables synchronous monitoring for mechanical‐electrical‐thermal parameters variations. Li et al.^[^ ^23^ ^]^ attached the FBG sensors to the cell surface to obtain additional process‐related signals to capture the temperature variations of multi‐point, which are employed as information features to co‐estimate SOC and SOH. Despite this, some limitations exist in practical applications, which are primarily caused by limited grating positions that can be detected. The number of grating regions must remain below a certain threshold to prevent measurement inaccuracies. While recent study^[^ ^24^ ^]^ achieved the etching of thousands of gratings on one single fiber, the increase in sensing capability has also led to problems such as manufacturing complexity and signal processing difficulties. Moreover, meticulous care is essential during the installation owing to their intrinsic fragility and vulnerability. The sensor performance is directly impaired by any damage to the grating area. Although multi‐grating FBGs may cost over $15, the enhanced accuracy and anti‐interference ability make them potentially indispensable for specific applications compared to conventional sensors.

Ordinary and FBG sensors have yielded certain achievements in thermal monitoring, but the solutions and effectiveness are often limited, which are inherently confined to discrete and single point measurement. Owing to the resolution constraints, some features may be missed to hinder complete representation of the spatial state, such as temperature distribution. The shortcomings obstruct comprehensive analysis of battery characteristics and reduce the effectiveness of management systems in optimizing performance and safety. How to break through the limitations of the number of measurement points and monitoring resolution is one of the thorny scientific challenges?

With technological advancements, high‐resolution distributed optical fiber sensors (DOFS) based on optical frequency domain reflectometry (OFDR) have been employed. Yu et al.^[^ ^25^ ^]^ utilized Rayleigh scattering‐based DOFS to accurately measure temperature variations in real time for A5‐sized (3.6Ah) pouch cell, which explored in‐plane temperature differences and the movement of the hottest regions. They pointed out that DOFS based on OFDR technology can provide higher distributed temperature measurement effect with improved accuracy compared to TCs. Under 10 °C and 5C conditions, the in‐plane temperature difference measured was 307% higher than that obtained by TCs. However, they did not further analyze the underlying mechanisms of battery heat generation and evolution. Full‐range surface measurement coverage was not achieved, and the complete surface temperature field distribution also could not be captured. Li et al.^[^ ^26^ ^]^ employed DOFS technology and cellophane fiber to monitor electrode strain in real time with high fidelity. Leveraging the advantages, they designed the dense sensing network and proposed a localized state of charge (SOC) visualization imaging method to observe evolution trends over time with an error below 2.01%. The research focused on the relationship between strain and SOC, isolating the temperature influence to prevent them from obtaining comprehensive thermal information. The line‐shaped sensor deployment also results in monitoring deficiencies and blank. Similarly, Marco et al.^[^ ^27^ ^]^ monitored the internal temperature of 21 700 battery with DOFS. They discussed the axial thermal distribution and hotspot migration within the cell. Unfortunately, the temperature measurement was limited in specific regions along the fiber length, and the acquisition of full‐range temperature distribution remains a crucial problem. Chen et al.^[^ ^28^ ^]^ investigated distributed temperature measurement of optical fiber arc discharge using OFDR technology. The maximum detected temperature reached 2100 °C, which confirmed the outstanding temperature sensing capability. They quantitatively analyzed the thermal sensitivity coefficient of single mode fibers using the correlation coefficient, laying the foundation for future studies.

The above‐mentioned studies collectively validate the effectiveness and feasibility of DOFS in battery thermal monitoring applications. Compared to FBGs, DOFS offers higher precision and faster response speed. However, due to factors such as deployment methods and manufacturing processes, it only enables continuous distributed measurements in localized areas, which fails to fully achieve the crossing from “point” to “surface” to cause certain limitations. How to achieve full‐range high‐precision temperature field monitoring over the entire surface is another formidable scientific challenge. Notably, an integrated “sensing + self‐healing + advanced BMS” paradigm is central to the advancement of next‐generation batteries, which relies on embedding multi‐modal sensors directly within the cell architecture, facilitating real‐time degradation diagnostics and repair mechanisms to substantially prolong the service life.^[^ ^29^ ^]^ Hence, there is increasing interest among researchers in deploying embedded OFS to accurately monitor the internal state and address safety concerns, whether in cylindrical 18 650 or pouch cells^[^ ^30^, ^31^, ^32^, ^33^, ^34^ ^]^ Unfortunately, the implantation of OFS remains a significant challenge that requires further investigation, which may lead to issues such as separator penetration, poor contact between the anode and cathode, and packaging perforation.^[^ ^35^, ^36^, ^37^, ^38^ ^]^ Eventually, the battery capacity and safety are significantly diminished to impact the normal operation. Fleming et al.^[^ ^35^ ^]^ placed thermal sensors between the anode and separator of pouch cells. However, cycling tests and post‐mortem analysis revealed that the direct implantation led to slight gaps between the anode and separator, resulting in capacity loss. Removing a portion of the electrode material to create space for sensor can help alleviate the uneven pressure. Lu et al.^[^ ^5^ ^]^ emphasized that the appropriate implanted location within the battery is critical for both protecting the sensor from damage and ensuring long‐term system stability. According to Jinasena et al.,^[^ ^36^ ^]^ the main challenge of implantation is to complete it without damaging the battery's anode, cathode, and electrolyte, which requires effective insulation, distributed measurement, high flexibility, and long‐term stability. Li et al.^[^ ^2^ ^]^ contended that one of the key factors for the OFS stable performance in LIB is proper and correct implantation. Initially, the consideration is typically to place the sensor between the electrodes before assembly. However, when transitioning to engineering applications, it is essential to optimize the distribution and installation within the electrodes to prevent capacity loss and prolong life. Improper embedding can easily lead to localized Li+ plating or even puncturing the separator, which increases the risk of thermal runaway and affects the safety. Hence, Li et al.^[^ ^22^ ^]^ placed two OFS into the specifically designed support beam and simulated the implantation to obtain strain and temperature information within the measurement unit. Similarly, Wang et al.^[^ ^37^ ^]^ designed a functional electrode integrated with OFS to address the above issues. However, the FE fabrication and operation are relatively complex. At present, most applications are still at laboratory stage and the practical feasibility remains to be assessed.

In this study, the DOFS based on OFDR technology serves as an advanced in‐situ method for precise temperature monitoring. Specifically, temperature‐DOFS (*T*‐DOFS) is gyrally adhered along the width of the high‐capacity pouch LIB used in BYD EVs, rather than embedding, which enables real‐time monitoring of in‐plane heat generation and temperature evolution under various current profiles. Further, we proposed the full‐range temperature reconstruction method based on locally adaptive radial basis function (LARBF) adjustment, which aims to compromise the global smoothness and local variability. Meanwhile, the uncertainty quantification (UQ) is incorporated to comprehensively assess the reliability and effectiveness of the results with confidence level. Different reconstruction results from two DOFS deployments are compared to explore the adaptability of this approach. Comparison with other six mainstream measurement methods, such as TCs, IT, FBG, and different shaped layout DOFS, also confirms the application potential of proposed method in battery thermal management systems. The merging of OFDR technology, LARBF interpolation‐reconstruction, and the UQ provides a comprehensive end‐to‐end solution for thermal monitoring of large‐format EVs pouch cells through novel integration, targeted adaptation, and extensive synergistic application, aiming to effectively overcome the limitations of conventional methods. Several key contributions are detailed as follows.

1. The practical DOFS spiral‐serpentine deployment configuration is presented to enable precise temperature distribution monitoring in large‐capacity pouch battery. It overcomes the limitations imposed by information gaps from discrete points, which also achieves the leap from point‐level sensing to comprehensive surface‐level measurement.
2. Systematic investigations are presented which aim to spatiotemporal evolution for heat generation under varying current rates. The underlying mechanism of temperature heterogeneity is illustrated to offer valuable insights and guidance for optimizing battery temperature uniformity.
3. The proposed high‐fidelity reconstruction method can accurately capture the variations in the full‐range temperature field without a complex neural network or significant memory consumption. UQ enhances the results' credibility with the maximum standard deviation (STD) below 0.3 °C. It demonstrates the exceptional potential of DOFS for tracking thermal anomalies and monitoring thermal states.

## 2 Experimental Results Analysis and Discussion

### 2.1 Surface Temperature Evolution by DOFS

**Figure 2:**
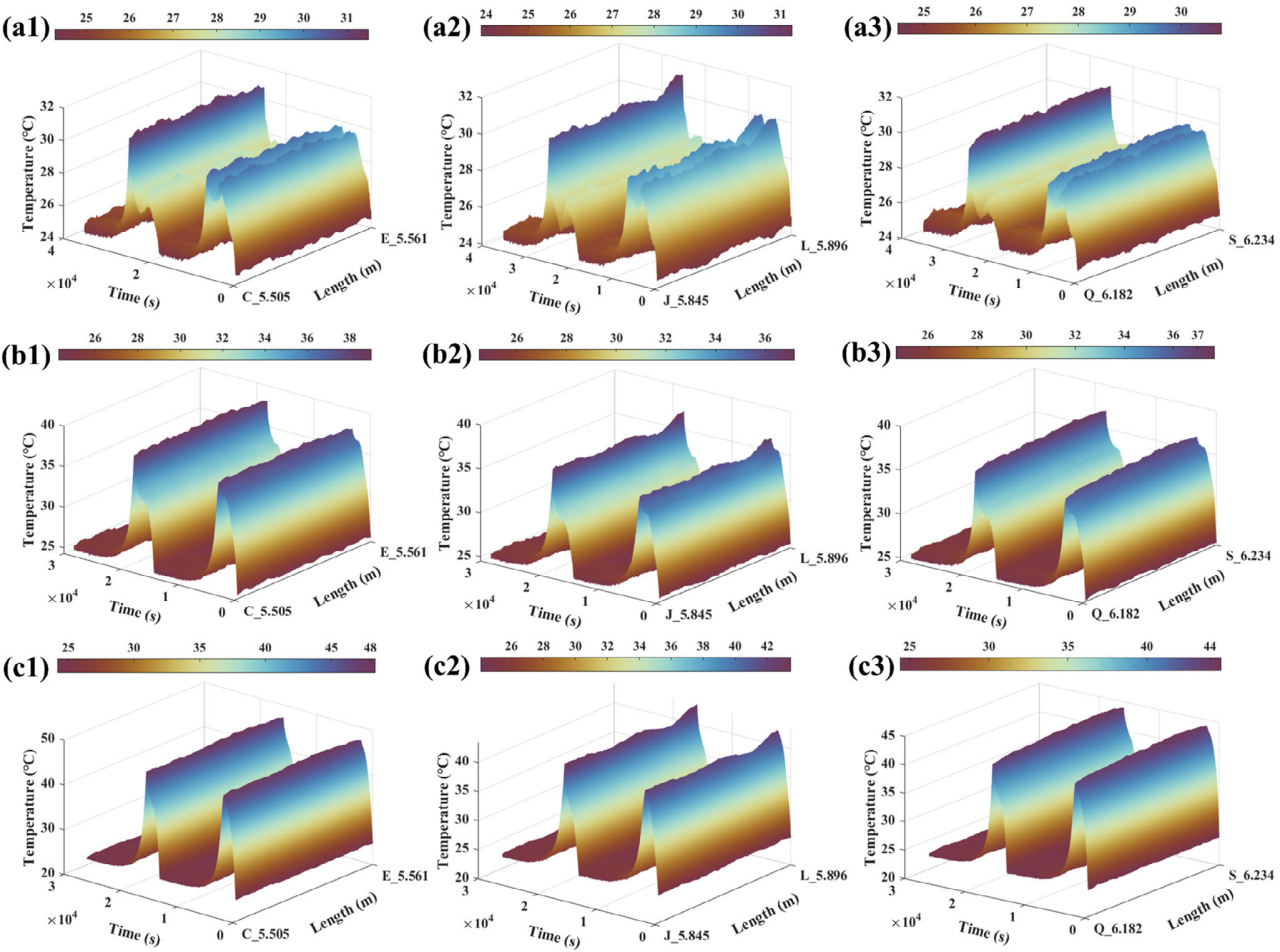
Surface temperature evolution under different current with various segments (a1) @0.5C CE (a2) @0.5C JL (a3) @0.5C QS (b1) @1.0C CE (b2) @1.0C JL (b3) @1.0C QS (c1) @1.5C CE (c2) @1.5C JL (c3) @1.5C QS.

**Figure** **2** illustrates the temperature evolutions along the battery width under different C‐rates with a serpentine‐spiral DOFS layout of 0.991 m total length, which is deployed along the width direction of the pouch cell. In all results, the *x*‐axis represents the optical fiber length corresponding to different measurement positions, as listed in **Table** **1**. The *y*‐axis denotes the charge‐discharge time. To reduce the redundancy and enhance clarity and readability, three representative segments (CE, JL, and QS) depicted in Figure 11 are selected to illustrate the temperature evolution. The results in these segments seem to be able to effectively represent the temperature variations across different regions of the cell surface (0<*x_α_* <99 mm, 240 mm<*y_α_* <320 mm; 0<*x_β_* <99 mm, 120 mm<*y_β_* <200 mm; 0<*x_γ_* <99 mm, 0 <*y_γ_* <80 mm).

**Table 1:** Measurement points calibration results under dual U‐shaped DOFS deployment.

Point Type	①	②	③	④	⑤	⑥	⑦	⑧
*P*_locating_ (m)	1.9	2.2	2.235	2.26	2.54	2.56	2.595	2.885
*P*_actual_ (m)	4.9	5.2	5.235	5.26	5.54	5.56	5.595	5.885

It can be seen from Figure 2 that different current loads lead to distinct temperature distributions and gradients, which further highlights the non‐uniformity of temperature distribution. At the beginning, the temperatures at all points remain stable around 25 °C, which is consistent with the ambient temperature. As charging proceeds, thermal gradients gradually emerge to lead to divergent changes across locations. Toward the end of the constant charging (CC) stage, the temperature peak is observed. Upon entering the constant voltage (CV) stage, the current decreases to cause either a second peak or decline. By the end of the rest, temperatures generally return to 25 °C except at a few specific points. As the current rate increases, the second peak progressively diminishes and merges into the first peak with the max value occurring at the end of the CC. We consider that heat conduction and thermal hysteresis are the main contributing factors. At low current rates, heat is generated, gradually accumulates, and transfers to the surface to form the first temperature peak. As charging progresses, the internal electrochemical reactions gradually reach equilibrium. However, due to the hysteresis effect, temperature changes lag behind current changes, which makes the second peak. In contrast, higher current rate accelerates internal electrochemical reactions and Li+ insertion/extraction to enhance the heat accumulation and conduction. Hence, the temperature exhibits a single peak.

At low current rates, as shown in Figure 2(a1–a3), the temperature evolution across the three regions is generally consistent with peak remaining below 32 °C, which indicates that heat generation is relatively uniform and poses minimal risk under low‐rate charging. Notably, the JL segment exhibits greater variability among each point to demonstrate stronger thermal heterogeneity within the central area, which is likely attributed to the combined effects of internal heat source distribution and external heat dissipation conditions. This phenomenon is mitigated but not eliminated as the current rate increases. Contrary to the findings reported in,^[^ ^38^ ^]^ the hottest regions do not appear randomly. As shown in Figure 2(b1–b3), when the current rises to 1C, temperature variations across segments become smoother. However, the peak in the CE segment is approximately 2 °C higher than that in the JL segment, which indicates that heat generation near the positive tab region (0<*x_α_* <99 mm, 240 mm<*y_α_* <320 mm) is more pronounced. This effect is further exacerbated with increasing current rates, as presented in Figure 2c.

At higher current rates (Figure 2c1–c3), the peak in the CE segment exceeds 47 °C, while those in the JL and QS segments remain below 43 and 44 °C, respectively. This discrepancy can perhaps be attributed to overpotential and Joule heating. During high‐rate charging, to drive the Li+ extraction‐intercalation and cross the electrolyte interface, both electrodes need to overcome the resistance of charge transfer and solid‐state diffusion, which is manifested as overpotential. The overpotential at the positive electrode during charging is much higher than that at the negative electrode to produce more concentrated Joule heat. Meanwhile, the resistivity of the aluminum tab is approximately 1.7 times that of copper, causing more heat to be generated at the positive tab under the same current. Previous studies^[^ ^39^ ^]^ also indicated that the overpotential primarily originates from ionic diffusion and charge transfer processes within the electrode solid phase and electrolyte. The cathode tab region exhibits a greater impedance gain to result in more significant heat generation. The above current‐dependent thermal behaviors highlight the necessity of adopting stage‐specific thermal management strategies to mitigate localized overheating risks.

### 2.2 Temperature Field Precise Reconstruction with Uncertainty Quantification

#### 2.2.1 Overall Process

**Figure 3:**
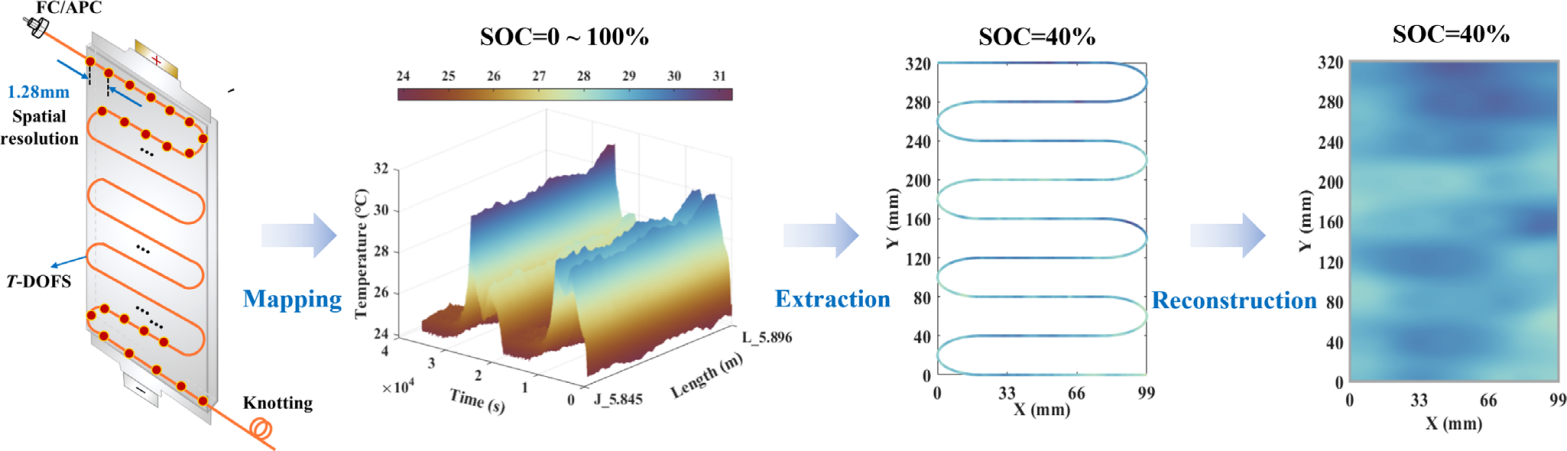
The overall process of temperature field reconstruction.

**Figure 4:**
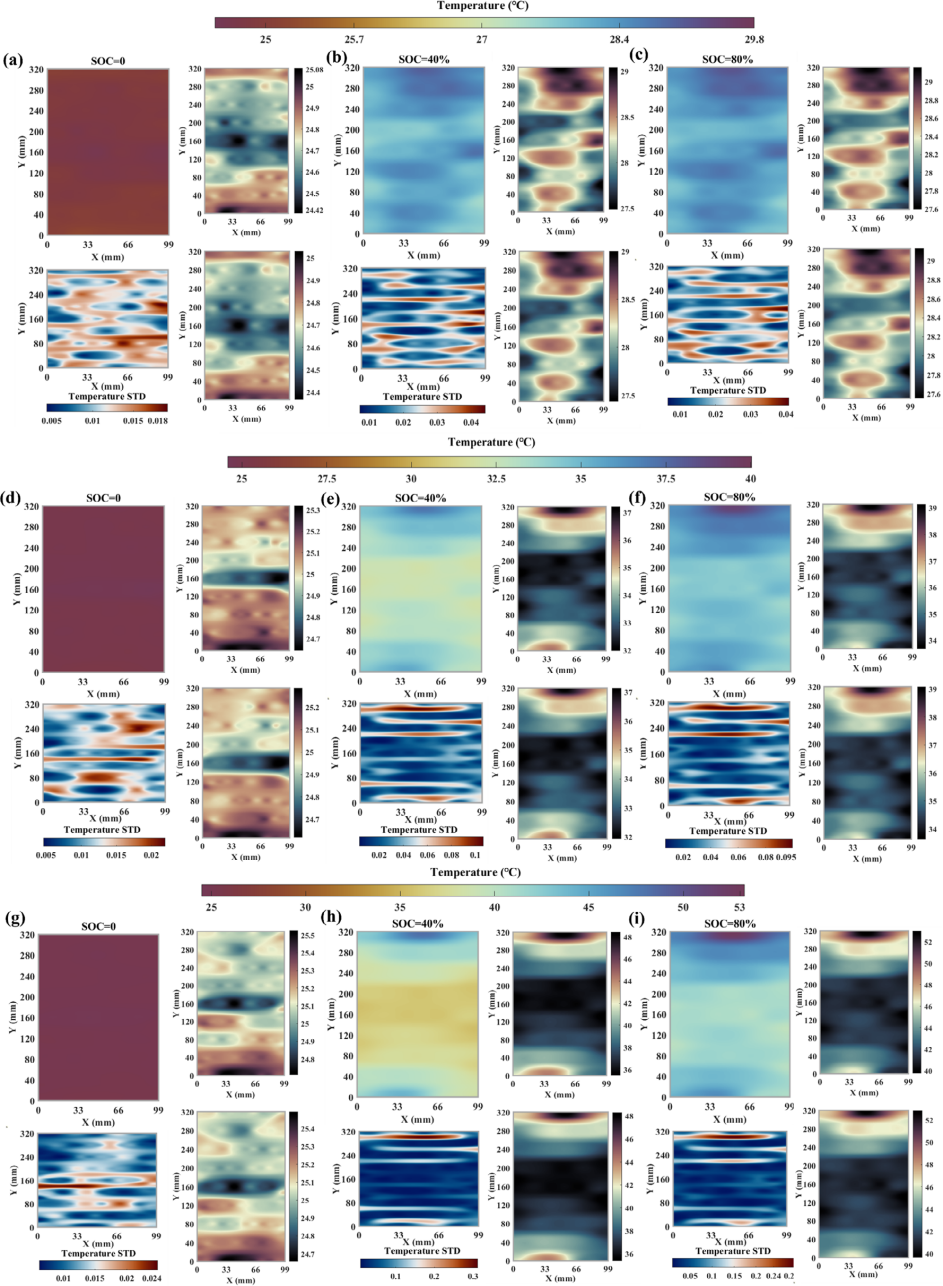
The temperature reconstruction results and error under 25 °C with the proposed layout. For each subplot group, the upper‐left shows the battery reconstructed surface temperature at the given SOC, the lower‐left depicts the reconstruction standard deviation, and the upper‐right and lower‐right display the visualized upper and lower bounds of the CI, respectively. (a) @0.5C SOC = 0 *t* = 0 (b) @0.5C SOC = 40% *t* = 2903s (c) @0.5C SOC = 80% *t* = 5705s (d) @1.0C SOC = 0 *t* = 0 (e) @1.0C SOC = 40% *t* = 1439s (f) 1.0C SOC = 80% *t* = 2877s (g) @1.5C SOC = 0 *t* = 0 (h) @1.5C SOC = 40% *t* = 961s (i) @1.5C SOC = 80% *t* = 1930s.

To characterize the surface temperature more clearly under different SOC and overall evolution process, we developed a high‐accuracy temperature field reconstruction method based on a Gaussian‐kernel locally adaptive RBF. Simultaneously, UQ is performed to assess the credibility of the interpolation results. The overall process is illustrated in **Figure** **3**, which consists of one‐dimensional data mapping, temperature information extraction under different SOC, and interpolation reconstruction. The specific implementation processes are presented in the Method section, which are not repeated here. The results are depicted in **Figure** **4**. For each subplot group, the upper‐left shows the battery reconstructed surface temperature at the given SOC. The lower‐left depicts the reconstruction STD that represents the uncertainty magnitude of predicted value. The upper‐right and lower‐right display the visualized upper and lower bounds of the confidence interval (CI) to help assess the results reliability, which implies a 95% probability of the true temperature lying within the interval. To reduce redundancy and enhance readability, results under three selected SOCs (0%, 40% and 80%) at various C‐rates are presented.

#### 2.2.2 Results and Analyses Under Spiral‐Serpentine Layout

Figure 4a–c presents the reconstructed temperature field, associated errors, and confidence intervals at 25 °C under 0.5C charging. As shown in Figure 4a, when SOC = 0, the surface temperature across the entire cell remains between 24.8 and 25 °C, with a maximum standard deviation below 0.018 °C. Upon charging to 40% SOC (2903 s), pronounced temperature nonuniformity emerges. Most areas exhibit temperatures between 27 and 28.4 °C, while localized regions (e.g., 33 mm<*x*<99 mm, 260 mm<*y*<320 mm) show elevated temperatures approaching 29 °C. As presented in Figure 4b, the temperature STD across most regions remains below 0.03 °C. Slightly larger deviations (>0.03 °C) are observed in localized areas (e.g., 66 mm<*x*<99 mm, 160 mm<*y*<200 mm; 0<*x*<33 mm, 70 mm<*y*<120 mm), which are likely attributable to the DOFS layout and cell packaging structures. The CI bounds range from approximately 27.5 –29 °C, confirming the accuracy of the reconstruction. As charging continues to 80% SOC (5705 s), the surface temperature further increases which accompanied by intensified nonuniformity. The STD distribution remains similar to that at 40% SOC, with a maximum value below 0.04 °C.

Figure 4d–f,g–i presents the reconstruction results and associated errors under 1C and 1.5C, respectively. As shown in Figure 4d, at SOC = 0, the cell surface temperature still remains around 25 °C, with most regions exhibiting STDs below 0.0125 °C. Relatively larger deviations (>0.015 °C) are observed in the upper right area (48 mm<*x*<99 mm, 220 mm<*y*<280 mm) and the central region (33 mm<*x*<66 mm, 60 mm<*y*<100 mm). Upon reaching 40% SOC (1439 s), further heat generation is observed, leading to an overall increase. The temperature within the region (0<*x*<99 mm, 60 mm<*y*<220 mm) rises above 32 °C. The temperature near the positive tab area is highest (≈37 °C), as confirmed by the CIs. Except for a few localized areas, the STDs remain below 0.04 °C. At 80% SOC (Figure 4f), heat generation and temperature nonuniformity further intensify. The max temperature reaches about 39 °C, and the overall STDs are less than 0.05 °C. The STDs are relatively larger but remain below 0.1 °C near the positive tab region (0<*x*<72 mm, 290 mm<*y*<320 mm). As the current rate increases, temperature nonuniformity under different SOCs becomes more pronounced, as shown in Figure 4g–i. The max value reaches approximately 52 °C. The STDs remain below 0.3 °C at 40% SOC (961 s) and below 0.28 °C at 80% SOC (1930 s), further confirming the effectiveness and reliability of the reconstruction method. Moreover, under all tested conditions, hotspots predominantly appear near the positive tab area, which highlights the necessity of employing strong adaptive measures such as localized cooling (e.g., air or liquid) to enhance the thermal regulation ability for a particular area.

### 2.3 Reconstruction Results Comparison of Different DOFS Layout

#### 2.3.1 Deployment and Calibration Instructions

**Figure 5:**
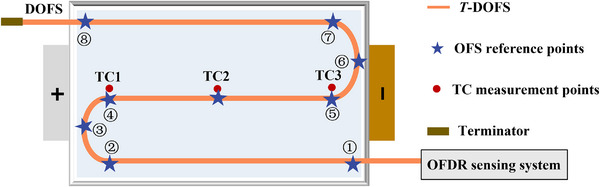
Schematic diagram of NCM battery setup with the dual U‐shaped DOFS.

**Figure 6:**
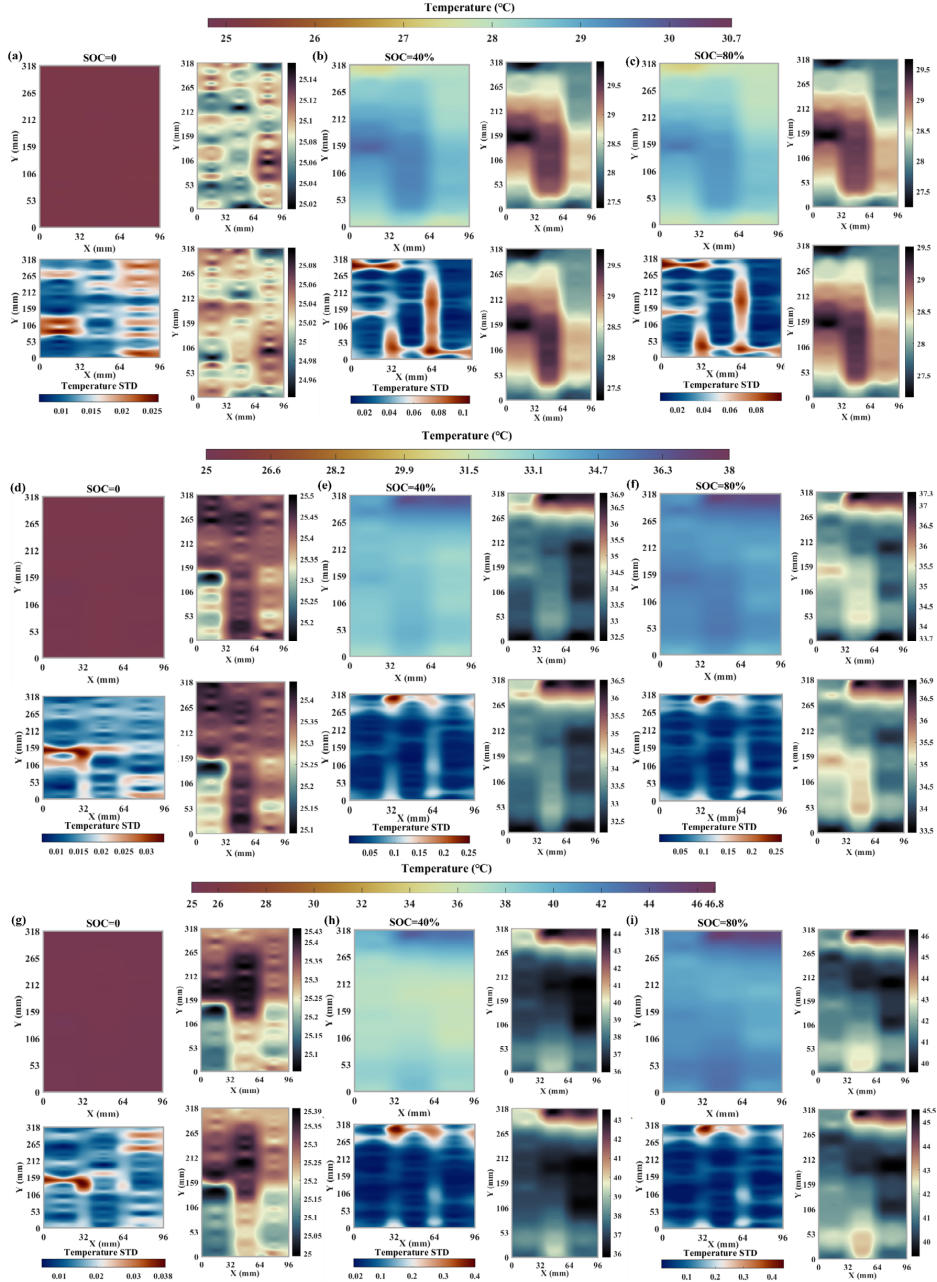
The temperature reconstruction results and error under 25 °C with dual‐U layout. For each subplot group, the upper‐left shows the battery reconstructed surface temperature at the given SOC, the lower‐left depicts the reconstruction standard deviation, and the upper‐right and lower‐right display the visualized upper and lower bounds of the CI, respectively. (a) @0.5C SOC = 0 *t* = 0 (b) @0.5C SOC = 40% *t* = 2835s (c) @0.5C SOC = 80% *t* = 5670s (d) @1.0C SOC = 0 *t* = 0 (e) @1.0C SOC = 40% *t* = 1418s (f) 1.0C SOC = 80% *t* = 2835s (g) @1.5C SOC = 0 *t* = 0 (h) @1.5C SOC = 40% *t* = 941s (i) @1.5C SOC = 80% *t* = 1883s.

In this section, the detailed comparisons are conducted between the proposed spiral‐serpentine shaped DOFS layout and our previously presented dual‐U layout^[^ ^40^ ^]^ (**Figure** **5**). Specifically, we provided the measurement points calibration results for the dual U‐shaped DOFS layout, as shown in Table 1. The total length of the *T*‐DOFS used is 0.985 m. Excluding minor positioning errors, a spatial discrepancy of approximately five measurement points (with a spatial resolution of 1.28 mm) is observed between the two layouts. We believe that this influence can be reasonably ignored to ensure the validity and reliability of the controlled experiment. The reconstruction results and error (0.5C, 1.0C, and 1.5C) with dual‐U layout are shown in **Figure** **6**.

#### 2.3.2 Results and Analyses Under Dual‐U Shaped Layout

As shown in Figure 6a, the cell surface temperature remains at 25 °C at 0.5C and SOC = 0, which exhibits excellent consistency and uniformity. It indicates that the resting time is sufficient to make strong consistency between surface temperature at any position and ambient temperature. The reconstructed STD is presented in the lower left corner of Figure 6a. The max surface temperature STD is less than 0.025 °C. Across most areas, STD remains stable within the range of 0–0.015 °C. In the localized region (0<*x*<32 mm, 54 mm<*y*<145 mm), a slightly elevated STD between 0.02 and 0.025 °C is observed. Meanwhile, in the cell right section (64<*x*<96 mm), the majority exhibits STD between 0.015 and 0.02 °C. These observations suggest that the cell surface is not perfectly uniform, which also means that minor temperature inconsistencies are present even under rest conditions. The CI upper and lower bounds are shown in the right half of Figure 6a. At a confidence level of 95%, the upper bound of the temperature distribution ranges approximately from 25.02 to 25.14 °C, while the lower bound ranges from 24.96 to 25.08 °C.

At 40% SOC (2835s), the reconstructed temperature field and STD are shown in Figure 6b. Overall, the left region (0<*x*<64 mm) exhibits higher temperatures with 28.5–30 °C. Due to the low current rate, temperatures near the negative tab (negative: 32<*x*<64 mm, 0<*y*<53 mm; positive: 32<*x*<64 mm, 265<*y*<318 mm) are comparable with the positive tab temperature being approximately 1 °C higher, which is consistent with previous findings. The max STD is 0.1 °C. Most regions show stable and accurate results with errors of 0.02–0.05 °C. Localized areas (28<*x*_a_<34 mm, 0<*y*_a_<106 mm; 60<*x*_b_<67 mm, 0<*y*_b_<260 mm; 0<*x*_c_<34 mm, 265<*y*_c_<318 mm) have larger deviations (0.08–0.12 °C). The STD is relatively larger near the negative tab area, which demonstrates that the temperature is higher than that near the positive tab at low current rates. At 80% SOC (5670 s), the reconstructed temperature field, as presented in Figure 6c shows no significant changes compared to 40% SOC. Both temperature distribution and STD remain similar. The upper and lower bounds of the 95% confidence interval are approximately 27.4–29.6 °C and 27.3–29.5 °C, respectively. Notably, under the unified color scale, the max surface temperature during 0.5C charging reaches about 30.7 °C.

Similarly, when the current is 1C and SOC = 0, the surface temperature remains at 25 °C as depicted in Figure 6d. Except for the certain region (32<*x*<64 mm, 106<*y*<159 mm) with a relatively large STD of 0.025–0.034 °C, the other region has low temperature STD below 0.02, which demonstrates the high accuracy of the proposed method. When the cell is charged to 40% SOC (*t* = 1418s), the temperature near the positive tab of approximately 36.7 °C is higher than that near the negative tab (≈33.1 °C). The corresponding temperature STD also illustrates this phenomenon. The positive tab region (32<*x*<64 mm, 265<*y*<318 mm) exhibits larger errors between 0.2 and 0.25 °C, while the negative tab area shows smaller STD. Elsewhere, the surface temperature remains around 31.5–33.1 °C. Although spatial inconsistency is evident, the thermal gradient is relatively small. Heat generation mainly concentrates in the positive tab and surrounding areas, which is further supported by the CI bounds indicating higher temperatures in this region.

As SOC reaches to 80% (2835s), the cell surface temperature further increases with most areas stabilizing around 35 °C, while that in positive and negative tab nearby regions rise to about 37 and 35 °C, as shown in Figure 6f. The temperature STD and CI remain similar to those at 40% SOC, so further details are omitted. Figure 6g–i presents the reconstruction results and error under 1.5C. The highest temperature reaches approximately 46 °C. At 40% and 80% SOC, the STDs are both below 0.4 °C with the distribution similar to that at 1.0C. Nevertheless, the temperature difference between the positive and negative tab regions is greater.

#### 2.3.3 Detailed Comparisons Between Two Deployment Schemes

**Figure 7:**
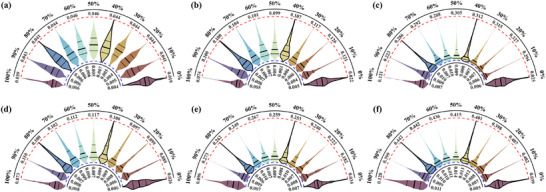
The violin plots of the reconstructed temperature STD under different DOFS layouts. (a)–(c) correspond to the proposed scheme, while (d)–(f) represent the scheme in ref.[40] Each subplot includes a total of 10 representative SOC levels ranging from 0% to 100%. (a) 0.5C with proposed layout (b) 1.0 C with proposed layout (c) 1.5C with proposed layout (d) 0.5C with dual‐U layout (e) 1.0 C with dual‐U layout (f) 1.5C with dual‐U layout.

Detailed comparisons are presented in **Table** **2** and **Figure** **7** to quantitatively assess the effectiveness and accuracy of the different layout schemes. As shown in Table 2, when employing the proposed spiral‐serpentine shaped DOFS layout, the temperature STDs under various current rates and SOCs are consistently lower than those obtained with the dual‐U layout. Most areas exhibit deviations under 0.1 °C, which further validates the effectiveness of the proposed method. Notably, the peak value at 1.5C reaches approximately 53 °C, about 13% higher than that reconstructed results by the dual‐U layout. It highlights that the enhanced area coverage provided by the spiral‐serpentine design enables more comprehensive and accurate temperature information, to offer valuable insights for optimizing future battery thermal management systems and strategies.

**Table 2:** The indicators comparison of temperature reconstruction in different layout schemes.

Layout type	Current	Max/min *T*	STD	CI upper bound	CI lower bound
**Proposed spiral‐serpentine shaped**	0.5C	29.8/24.7	0.005–0.018(SOC = 0)	24.42–25.08	24.4–25.1
			0.01–0.042(SOC = 40%)	27.5–29	27.5–29
			0.008–0.04(SOC = 80%)	27.6–29.3	27.6–29.2
	1.0C	40–24.9	0.005–0.021(SOC = 0)	24.7–25.3	24.65–25.25
			0.01–0.1(SOC = 40%)	32–37	32–37
			0.01–0.095 (SOC = 80%)	34–39	33.9–39
	1.5C	53–24.9	0.006–0.024(SOC = 0)	24.7–25.5	24.7–25.45
			0.05–0.3(SOC = 40%)	36–48	36–48
			0.03–0.28 (SOC = 80%)	40–52.3	40–52.2
Dual U‐shaped	0.5C	30.7/24.9	0.0075–0.025(SOC = 0)	25.02–25.15	24.95–25.09
			0.015–0.11(SOC = 40%)	27.5–29.8	27.4–29.6
			0.01–0.1 (SOC = 80%)	27.4–29.6	27.3–29.5
	1.0C	38/25	0.007–0.034(SOC = 0)	25.15–25.5	25.1–25.44
			0.03–0.25(SOC = 40%)	32.5–36.9	32.3–36.5
			0.025–0.25 (SOC = 80%)	33.7–37.3	33.5–36.9
	1.5C	46.8/25	0.006–0.038(SOC = 0)	25.05–25.43	25–25.39
			0.02–0.4(SOC = 40%)	36–44	36–43.5
			0.02–0.42 (SOC = 80%)	39.8–46.2	39.5–45.5

Figure 7 presents detailed violin plots of the reconstructed temperature STDs under different current rates for the two DOFS deployment schemes. It can be seen that the proposed method consistently yields smaller reconstruction STDs across different current rates, with the maximum error remaining below 0.33 °C (at 1.5C, 40% and 80% SOC), demonstrating the effectiveness and superiority. Taking the 0.5C‐rate as an example, when SOC = 0, the violin plots appear “short and fat” (according to STD range) with low median values under both deployment schemes. Notably, the spiral‐serpentine shaped deployment results in smaller medians and quartiles. The overall distribution is highly concentrated, with most STD values below 0.018. It indicates that the surface temperature field is remarkably uniform at the onset of charging, exhibiting negligible thermal gradients. Under such conditions, our reconstruction model can confidently predict the temperature with extremely low uncertainty.

Between SOC = 10% and 40%, the violin plots become lanky, and the median value rises significantly to approximately 0.018. The STD distribution widens, and the pronounced upper “tail” emerges, which means that the regions with higher uncertainty are beginning to emerge. It suggests that as the charge/discharge process progresses, electrochemical reactions inside the battery become more active, leading to uneven heat generation. Thus, thermal gradients gradually develop to make an overall increase in prediction uncertainty. When SOC = 50% to 90%, the distribution enters the plateau phase, with the median remaining stable at around 0.022 and showing minimal fluctuation. The violin plots maintain a slender shape with an extended upper tail, indicating the continued presence of regions with high uncertainty. Notably, at SOC = 80%, the cell electrochemical activity reaches its peak, corresponding to the highest level of uncertainty.

At SOC = 100%, the violin plot visibly contracts and narrows, with the median dropping to 0.012 and the upper tail shortening accordingly. The overall distribution shifts back toward lower STD values. This phenomenon is consistently observed across all subplots. It reflects the slowdown of electrochemical reactions and the associated reduction in heat generation as the charging process nears completion. Internal heat begins to redistribute through conduction to make a gradual homogenization of the temperature field, with the weakened thermal gradients. Hence, the temperature field becomes “simple”, and the prediction uncertainty also significantly decreases accordingly. Moreover, these results clearly demonstrate the significant influence of current rate on the temperature distribution. As the current increases, the violin plots at high SOC levels become more elongated, as shown in Figure 7b,c,e,f with the most pronounced phenomenon observed under the 1.5C condition. It indicates that intensified heat generation at higher current rates, causes steeper thermal gradients and further exacerbates the temperature non‐uniformity and inconsistency.

### 2.4 Comparisons with Infrared Thermography

**Figure 8:**
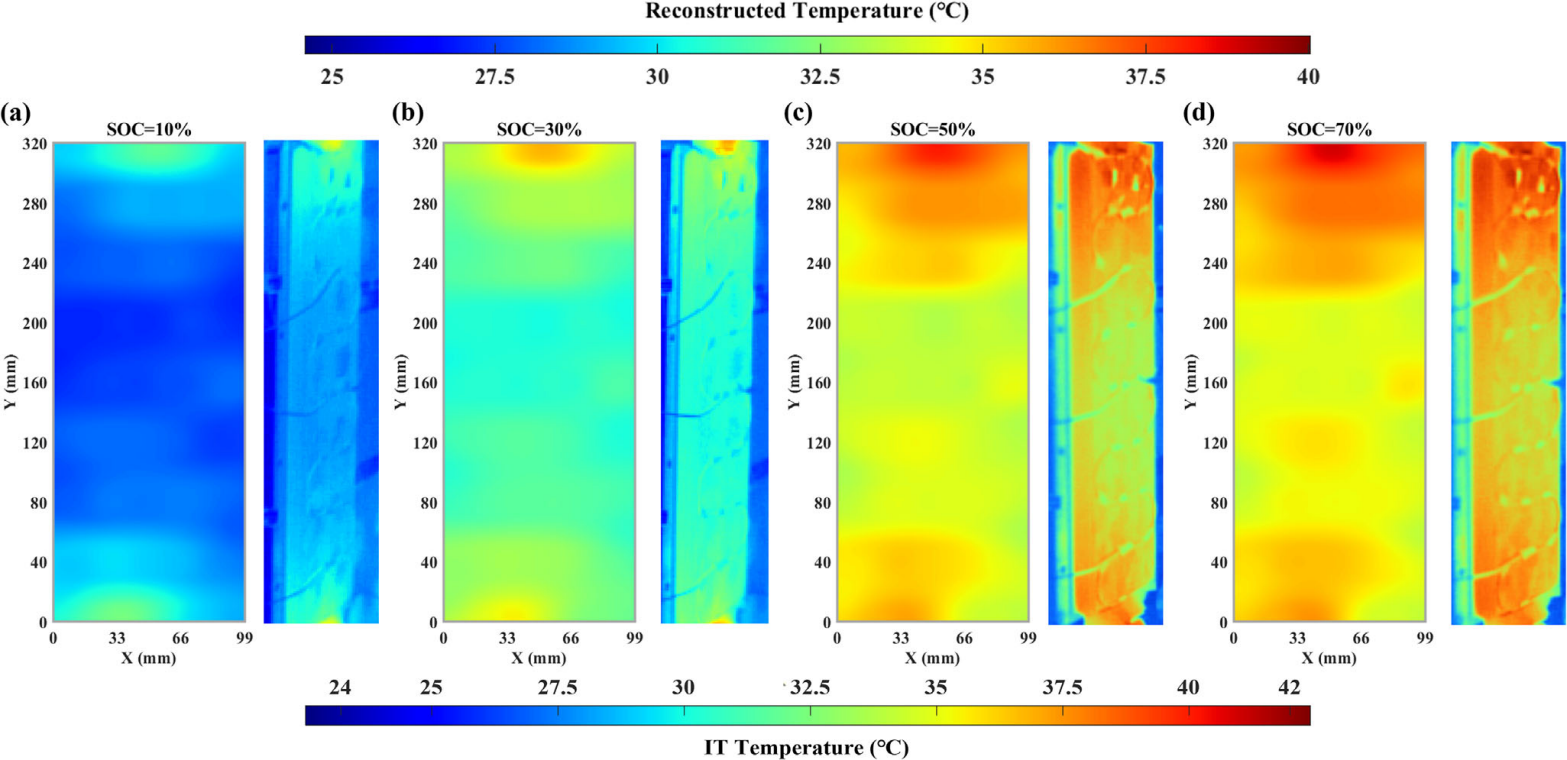
Comparison of reconstructed and actual temperature under 25 °C and 1C (a) SOC = 10% (b) SOC = 30% (c) SOC = 50% (d) SOC = 70%. Each subgraph contains the reconstruction result (left) and the true result (right).

To further demonstrate the effectiveness and reliability of the proposed method, the comparative experiments are conducted against infrared thermography (IT), which serves as a widely accepted reference technique for non‐contact temperature measurement. As shown in Figure 12, the IT is utilized to capture cell surface temperature under identical ambient conditions (@25 °C 0.5/1.0/1.5C). In this section, the Ti32 IT, manufactured by Fluke (USA), is employed for experimental measurements. The temperature measurement range is −20– 600 °C with the accuracy of ±2 °C and a thermal sensitivity of less than 0.045 °C. To enhance the clarity and readability, **Figure** **8** presents the comparisons under 1C with selected SOC characteristic points. It is worth noting that the reconstructed temperature results are visualized using the same color gradient scheme as the Ti32.

As shown in Figure 8a,b, at low SOC, the reconstructed temperature closely matches the actual battery temperature, with a deviation of approximately 1 °C. As SOC increases, as depicted in Figure 8c,d, heat generation intensifies in both the anode and cathode regions, and the temperature variation becomes more pronounced compared to the central area. The reconstructed temperature near the tab is higher than that measured by IT, and the identified heated region is also larger. The error exceeds 1 °C, which may be attributed to the limited resolution and accuracy of IT, resulting in potential measurement errors or thermal detail loss. Overall, the proposed DOFS‐based temperature reconstruction method provides a reliable representation of the battery surface temperature. The reconstructed temperature exhibits a trend consistent with IT measurements.

### 2.5 Comparisons with Different Measurement Methods

Seven commonly used temperature measurement methods are introduced for comprehensive comparison and analysis to further stress the advantages of the proposed methods. **Table** **3** presents the detailed contrast in accuracy, mode, reconfigurability, and adaptability. In Refs. [9] and,^[^ ^41^ ^]^ the TC with the accuracy of ±1 °C is utilized to monitor the temperature, which aims to establish model or estimate state. TC is flexible installation, user‐friendly and easy to operate, but it is prone to interference from the environment and other factors. FBGs are employed to obtain temperature information in ref. [21, 23] which have good repeatability and accuracy without electromagnetic interference affection, to suitable for complex environment. Careful handling is needed in operation process to avoid the grating region damage. Additionally, the limitation of number of discrete points is inherently existed, which hinders the complete representation of the system state. Dileep et al.^[^ ^42^ ^]^ systematically investigate the thermal responses of LIBs with different capacities under various charge/discharge rates and ambient temperatures using IT. Their findings provide key insights into hotspot localization and temperature gradient formation. However, despite the advantages of IT in terms of real‐time and non‐contact measurement, it suffers from relatively low accuracy and resolution, which loses the temperature details. Moreover, it is highly susceptible to environmental and human factors to cause certain limitations. In ref.,^[^ ^43^ ^]^ the simple line‐shaped DOFS is utilized to monitor temperature of 18 650 LIBs to estimate SOC. The newly sensed information can accurately reflect the heat generation rate in real time. However, although DOFS is deployed, it cannot overcome the point‐to‐surface limitation with only continuous measurement in local area. Meanwhile, it is noteworthy that the temperature cannot be reconstructed by using only TCs. When the FBGs are utilized, the temperature reconfigurability is also limited by the number of points and arrangement of optical fiber. As many grating regions as possible can improve the accuracy of the reconstructed field to a certain extent, but it will increase the related burden. Although the linearly arranged DOFS can achieve temperature reconstruction, its application is restricted to specific areas near the straight line, typically within a few millimeters around the measurement point. To solve the above problems, Zhang et al.^[^ ^40^ ^]^ introduce the U‐shaped layout DOFS in the recent research, enabling continuous measurement along the cell length direction. Nevertheless, the fiber coverage in this scheme remains incomplete along cell width, which may cause an inaccurate reconstructed temperature field. Similarly, in the latest ref.[30] the DOFS is arranged in an Archimedean spiral to monitor the temperature of small size cell. Unfortunately, owing to the inherent geometric characteristics, the monitoring regions are confined to specific area centered on the cell, which may cause information loss. Although they claim to achieve surface temperature monitoring, its feasibility and effectiveness in large‐format LIBs may require further evaluation. In addition, the vortex configuration also increases the complexity of operation, orientation, and demodulation.

**Table 3:** Comprehensive comparison of different measurement techniques.

Method	Accuracy	Mode	Reconfigurability	Adaptability
TCs,^[^ ^9^, ^41^ ^]^	±1 °C	Discrete point (single)	No	Flexible installation, user‐friendly, prone to interference
IT^[^ ^42^ ^]^	±2 °C	2D image	–	Real‐time, non‐contact, prone to losing details
FBGs,^[^ ^21^, ^23^ ^]^	±0.1 °C	Discrete point (single/multiple)	Limited to a number of points	Suitable for complex environments
Line‐shaped layout DOFS based on OFDR^[^ ^43^ ^]^	±0.1–0.4 °C	Continuous point	Specific area near the straight line	
U‐shaped layout DOFS based on OFDR^[^ ^40^ ^]^	±0.1–0.4 °C	Continuous (along the cell length)	Specific area near the U‐shaped line	Strong adaptability, anti‐interference capability
Vortex‐shaped layout DOFS based on OFDR^[^ ^30^ ^]^	–	Continuous (Archimedean spiral)	Entire surface (Inaccurate)	
Proposed serpentine‐spiral layout DOFS	±0.1–0.4 °C	Continuous (along the cell width)	Entire surface (High‐fidelity, Accurate)	

In this paper, the proposed *T*‐DOFS measurement approach, implemented by serpentine‐spiral arrangement, allows continuous temperature measurement with accuracy of ±0.1 °C across the entire battery surface. Further, the enhanced measurement precision and spatial resolution (1.28 mm) allow for accurate and high‐fidelity reconstruction of the temperature distribution using spatial mapping and interpolation algorithms such as proposed adaptive Gaussian kernel‐based RBF with uncertainty quantification, which provides rich information for thermal management. Compared to the aforementioned methods, it bridges the gap between point and surface measurements, effectively mitigating information loss and locality issues while demonstrating superior adaptability and anti‐interference capability. More importantly, it can be easily extended to other types of batteries and offers valuable guidance for future research on advanced sensor embedding.

### 2.6 Thermal Management Optimization Strategy

The above experimental results highlight a significant thermal management challenge in pouch LIBs with their inherent structure and characteristics: temperature uniformity deteriorates markedly with increasing C‐rates. The area near positive tab is identified as the primary hotspot, with its temperature escalating as the current increased. The positive and negative tabs, along with their adjacent regions, serve as primary current collection areas, which exhibit significantly higher Ohmic heating and contact thermal resistance compared to other locations. It promotes the formation of localized hotspots, which in turn accelerate corresponding degradation and elevate the potential for thermal runaway. The combination of spiral‐serpentine DOFS deployment and high‐precision reconstruction algorithm facilitates the accurate acquisition of full‐field temperature distribution and hotspots, which is essential for developing robust thermal management strategies to ensure system safety, stability, and efficiency.

Specifically, three aspects are encompassed in optimization strategy in our opinion. First, at the source level, heat generation is suppressed through geometric and material enhancements. The cross‐sectional area of the tab can be appropriately increased within the spatial permission to reduce its inherent resistance and decrease Joule heat. Concurrently, advanced connection technologies such as laser welding can reduce interfacial resistance and achieve higher consistency. Secondly, systematically enhance the thermal conduction pathway. The core idea is to establish an efficient “heat highway” to reduce temperature non‐uniformity. Thermal interface materials with high adaptability and conductivity, such as thixotropic thermal gel and flexible pyrolytic graphite sheets, can be applied to optimize the high contact thermal resistance caused by microscopic air gaps and convert localized heat spots into more diffuse surface heat flux. Additionally, measures such as localized forced‐air cooling over tab regions and the attachment of phase change materials can also reduce temperature inconsistency and non‐uniformity. Through integrated optimization of the above multi‐dimensional strategies, it can effectively suppress the peak temperature at the tab region and minimize the thermal gradient (Δ*T*) across the cell surface. The uniformity of the cell thermal field can be improved. Further, it can provide valuable insights and guidance for the thermal management of modules and packs to reliably ensure the system operation safety and extend life.

## 3 Method

### 3.1 OFDR Technology Based on Rayleigh Scattering

**Figure 9:**
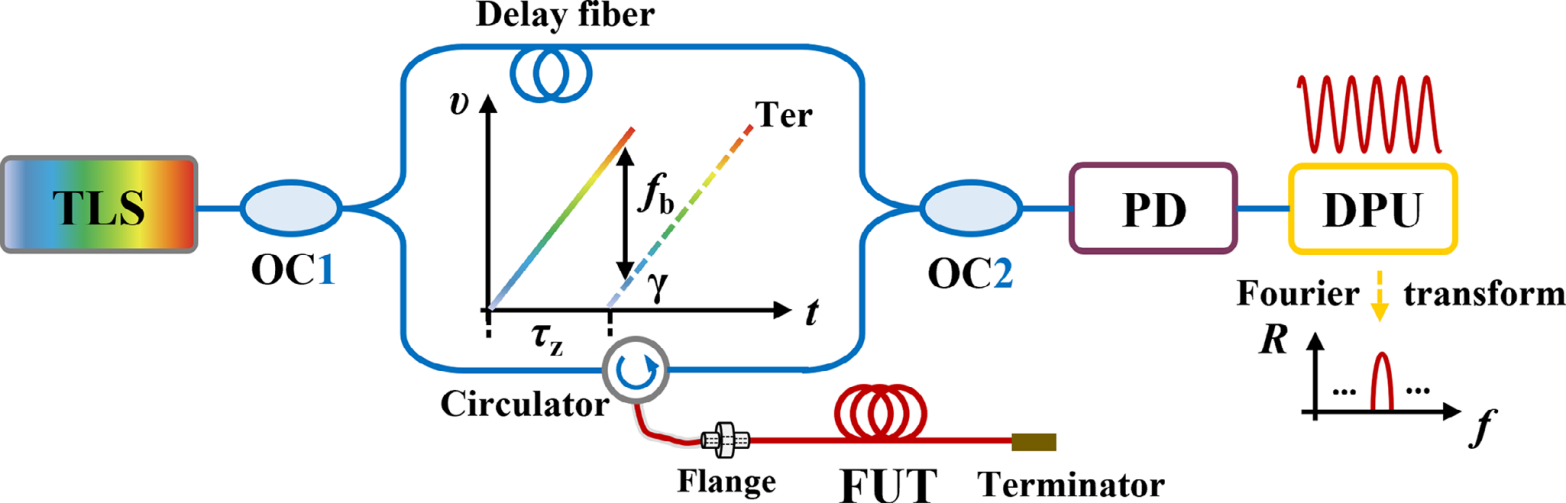
The primary compositions and working principle of OFDR.

In this study, the temperature measurement is achieved using DOFS based on OFDR technology. OFDR is an advanced measurement technique that operates by detecting the frequency shift of Rayleigh scattering signals generated by modulated probe light. The frequency spectrum of the interference signal reveals reflection peaks at different positions using Fourier transform. The beat frequency is proportional to the length of the optical fiber path, which enables distributed thermal or mechanical measurements at specific locations or along the entire fiber length. The compositions and principles are illustrated in **Figure** **9**.

As illustrated in Figure 9, the primary components of OFDR include the tunable laser source (TLS), a Michelson or Mach–Zehnder interferometer, an optical coupler (OC), a photodetector (PD), and data processing unit (DPU). TLS emits a continuous wave with its optical frequency linearly swept over certain range, typically spanning tens of nanometers. The swept light is divided into two paths by OC. One is directed through the reference arm, which acts as a local oscillator. The other is through the test arm, which contains the fiber under test (FUT). Within the FUT, light experiences Rayleigh backscattering due to imperfections or discontinuities and forms interference light with the reflected light. The IL is detected by the PD and subsequently analyzed, processed, and stored in DPU to extract the distributed sensing information.

It is assumed that the TLS is linearly tuned, which simplifies the calculation of the Rayleigh backscattering signals. The angular frequency can be expressed as Equation (1).

(1) $$\omega \left(t\right)=2\pi \left(f_{0}+\gamma t\right)$$

where *f*_0_ is the initial frequency of the light source, *γ* is the tuning rate. Hence, the optical phase for performing linear optical frequency tuning is as follows.

(2) $$\varphi \left(t\right)=\int_{0}^{t}\omega (t)dt+\varphi_{0}=2\pi f_{0}t+\pi \gamma t^{2}+\varphi_{0}$$

where *φ*_0_ is the initial phase. In actual conditions, the light source exhibits a phase term *ϕ* that fluctuates randomly with time. The above equation becomes Equation (3).

(3) $$\varphi \left(t\right)=2\pi f_{0}t+\pi \gamma t^{2}+\phi \left(t\right)$$

Then, the electrical field at the reference light can be expressed as *E_r_* (*t*).

(4) $$E_{r}\left(t\right)=E_{0}\cdot e^{\left\{j[2\pi f_{0}t+\pi \gamma t^{2}+\phi \left(t\right)]\right\}}$$

where *E*_0_ is the initial amplitude. For the Rayleigh backscattering light, the electrical field can be expressed as Equation (5).

(5) $$\begin{array}{c} E_{s}\left(t\right)={\left\{R\left(\tau_{z}\right)\right\}}^{1/2}\cdot E_{r}\left(t-\tau_{z}\right)\\ ={\left\{R\left(\tau_{z}\right)\right\}}^{1/2}\cdot E_{0}\cdot e^{\left\{j\left[2\pi f_{0}\left(t-\tau_{z}\right)+\pi \gamma {\left(t-\tau_{z}\right)}^{2}+\phi \left(t-\tau_{z}\right)\right]\right\}}\\ ={\left\{r\left(\tau_{z}\right)\cdot e^{-\alpha \tau_{z}c/n}\right\}}^{1/2}\cdot E_{0}\cdot e^{\left\{j\left[2\pi f_{0}\left(t-\tau_{z}\right)+\pi \gamma {\left(t-\tau_{z}\right)}^{2}+\phi \left(t-\tau_{z}\right)\right]\right\}} \end{array}$$

where *R*(*τ*_z_) = *r*(*τ*_z_)·exp(‐*ατ*_z_ *c*/*n*) represents the reflectivity of the optical fiber. *τ*_z_ = 2 *Zn*/*c* is the time delay. *Z* is the distance from the position where the delay time is 0 to a certain measurement point. *n*, 𝑐, 𝛼 are the effective refractive index, the speed of light in a vacuum, and the attenuation coefficient, respectively.

The expression for the beat frequency signal resulted from the interference is shown in Equation (6).

(6) $$\begin{array}{ccc} I(t) & = & {\left|E_{r}\left(t\right)+E_{s}\left(t\right)\right|}^{2}=\left[E_{r}\left(t\right)+E_{s}\left(t\right)\right]{\left[E_{r}\left(t\right)+E_{s}\left(t\right)\right]}^{*}\\ & = & E_{0}^{2}\{1+R\left(\tau_{z}\right)+2\sqrt{R(\tau_{z})}\cos[2\pi (f_{0}\tau_{z}+f_{b}t-\frac{1}{2}\gamma \tau_{z}^{2}\\ & & +\phi \left(t\right)-\phi \left(t-\tau_{z}\right))]\} \end{array}$$

where *f_b_* = *γτ = 2nzγ*/*c* represents the frequency of the generated beat frequency signal. *ϕ*(*t*)‐*ϕ*(*t*‐*τ*_z_) is the phase noise term. By extracting the spectral shift, information regarding changes in temperature, strain, or other parameters can be obtained.

### 3.2 Temperature Measurement Based on OFDR

As mentioned above, the formed signal with a specific frequency directly corresponds to the certain measurement points on FUT. Therefore, the spatial resolution Δ*λ* can be presented by Equation (7).

(7) Δλ=cc2nofΔvsweep2nofΔvsweep

where *c* is the light speed in vacuum, *n*_of_ is the refractive index of the optical fiber, and Δν_sweep_ is the frequency range of TLS. In this study, *c* ≈ 3 × 10^8^m/s, *n*_of_ ≈ 1.46, the max value of supported Δν_sweep_ in our device is ≈160 GHz.

By merging multiple Δ𝑧 points, the resolution can be arbitrarily set. According to Equation (7), the minimum permissible Δ*λ* for our device is 0.64 mm, i.e., a measurement point positioned every 1.28 mm. However, we contend that adopting a spatial resolution below 1 mm could produce excessive data volume, thus imposing a substantial processing burden. Hence, the Δ*λ* is set to 1.28 mm in this paper to compromise calculation and accuracy. When the optical fiber is subjected to temperature or strain variations, its effective refractive index and length change accordingly, which always causes the wavelength shift in the Rayleigh scattering within the fiber. This relationship can be expressed as follows.

(8) ΔγΔγγ0γ0=KTΔT+KσΔσ

where Δ*γ* represents the frequency shift. *K_T_* = *α*+*δ* and *K_σ_* = 1‐*p_e_* are the temperature and strain sensitivity coefficients, respectively. *α* and *δ* are the thermal expansion and thermo‐optic coefficients, respectively. *p_e_* is the photoelastic coefficient. Δ*T* and Δ*σ* are the variations of temperature and strain.

**Figure 10:**
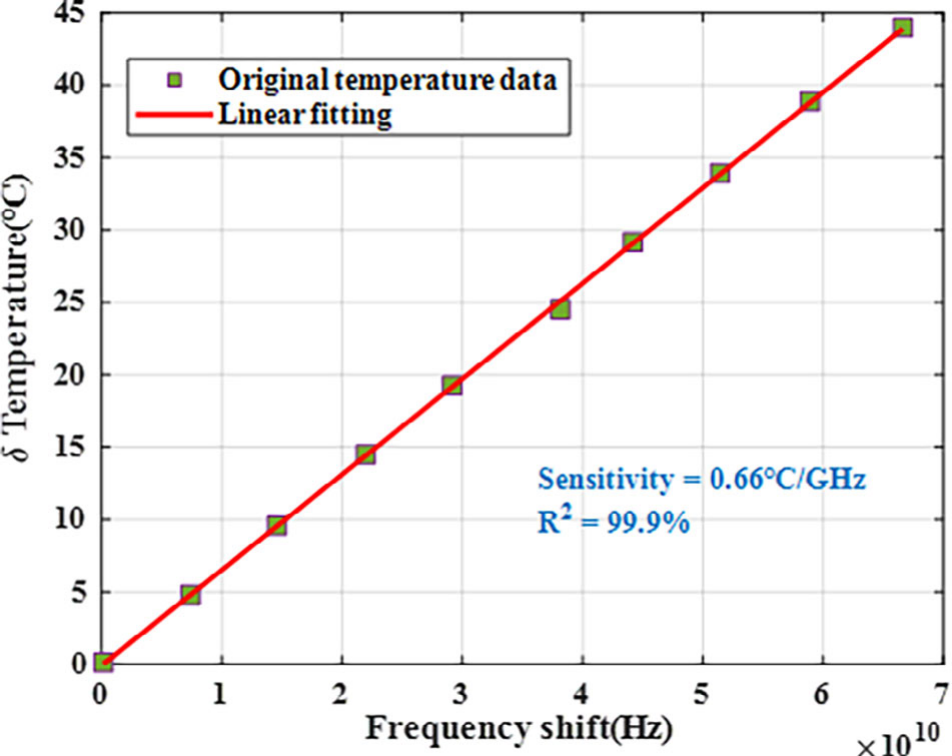
Temperature calibration results as a function of frequency shift with linear fitting.

In this study, the control variable method is employed to separate the effects of temperature and strain. The *T*‐DOFS is fabricated by encasing a 165 µm diameter polyimide (PI)‐coated optical fiber into PFA tube to effectively shield external stress. The selected PFA material exhibits characteristics such as high temperature resistance, efficient heat transfer, and strong toughness. The outer diameter is 1.15 ± 0.05 mm, the wall thickness is 0.15 ± 0.05 mm, the minimum bending radius is 15 mm, and its operating temperature ranges from −60 °C to +250 °C. The optical fiber is loosely inserted into the PFA tube, which ensures that the sensor remains sensitive to temperature changes without being affected by mechanical deformation. All optical fiber sensors are rigorously calibrated and screened prior to delivery. **Figure** **10** presents the temperature calibration results of *T‐*DOFS. The detailed procedures can be found in ref. [25] which are not elaborated here.

### 3.3 1D Data Mapping

*T*‐DOFS is affixed along a predefined path on the surface to provide continuous thermal monitoring. The raw measurements are recorded as 1D data pairs (*L_i_*, *T_i_*), where *L_i_* denotes the cumulative length along the optical fiber from its origin and *T_i_* is the corresponding temperature. We transform each 1D measurement into a 2D point (*x_i_*, *y_i_*, *T_i_*), which corresponds to physical location and temperature on the surface. Feature points such as turning points and segment junctions serve as spatial anchors to define the optical fiber trajectory, which provide geometric constraints that allow for precise transformation from optical fiber length‐based indexing to 2D spatial representation. Once the feature points are established, the path is decomposed several discrete straight‐line and semi‐circular arc segments, enabling a structured mapping of temperature data to the battery surface.

For straight lines, consider an optical fiber segment of length *O*_MN_ connecting the points *P*_M_ (*x*_m_, *y*_m_) and *P*_N_ (*x*_n_, *y*_n_). The coordinate (*x_i_*_1_, *y_i_*_1_) of the point at distance *l*∈[0, *O*_MN_] is calculated by Equations (9) and (10).

(9) $$x_{i1}=x_{m}+\frac{l}{O_{\mathrm{MN}}}\left(x_{n}-x_{m}\right)$$

(10) $$y_{i1}=y_{m}+\frac{l}{O_{\mathrm{MN}}}\left(y_{n}-y_{m}\right)$$

For semi‐circular arc segments, the curvature‐based mapping method is adopted. The optical fiber layout is characterized by a known radius *R* and central angle *θ*, determined by the feature points at the arc endpoints. The temperature measurement positions along the arc are computed based on relative arc lengths and then mapped to the 2D plane using a polar‐to‐Cartesian transformation.

(11) $$\left(x_{i2},y_{i2}\right)=\left(x_{o},y_{o}\right)+R\cdot \left(\cos\theta_{i2},\sin\theta_{i2}\right)$$

where (*x*_o_, *y*_o_) is the center coordinate of semi‐circular arc, *R* represents the radius, *θ* is the central angle of the circle. By the above mapping process, all temperature information along the optical fiber is accurately mapped onto the 2D spatial domain, thus preserving the high fidelity of the sensing‐measuring configuration.

### 3.4 Temperature Information Extraction Under Different SOC

In this study, under different current rates, the battery undergoes a complete charge–discharge cyclic test. The employed battery tester with corresponding host computer is capable of recording the instantaneous charge/discharge capacity *Q_t_* and the corresponding maximum charge/discharge capacity *Q*_max_ at each time point. Hence, the current SOC value for charging/ discharging can be calculated using Equation (12).

(12) $$\begin{array}{c} SOC_{t,\mathrm{charge}}=\frac{Q_{t}}{Q_{\max}}\times 100\%\\ SOC_{t,\mathrm{discharge}}=\left(1-\frac{Q_{t}}{Q_{\max}}\right)\times 100\% \end{array}$$

Subsequently, the time intervals corresponding to different SOC segments, such as [*t*_1_, *t*_2_] can also be obtained from the data recorded by the host computer testing software. Based on these time intervals, the temperature data acquired by the *T*‐DOFS can be matched, enabling the extraction of fiber temperature data within different SOC ranges. Notably, it is essential to ensure that the timestamps of the host computer in the distributed fiber sensing system and the battery testing system are synchronized. The sampling intervals of both systems must be consistent.

### 3.5 Locally Adaptive Radial Basis Function Interpolation

The temperature reconstruction from DOFS data points requires an interpolation process that maps the scattered measurements to the continuous spatial domain. Hence, a high‐resolution grid is first established on the surface to provide a structured framework. The surface is covered by a grid with 2 mm spatial resolution to ensure all points within the sensing area are captured. Grid nodes represent positions where temperature values are to be estimated, allowing for a seamless transition between discrete sensor readings and a continuous temperature distribution across the battery surface. Then, the DOFS data points are mapped to the structural domain with interpolation method.

Radial basis function (RBF) is a commonly used interpolation method that is widely applied for the smooth reconstruction of scattered data. Its core concept is to employ distance‐dependent basis functions to construct continuous interpolation functions that approximate or reconstruct the target field. It works by estimating the temperature at any given grid point as a weighted sum of the measurement value at the sensor locations. The weight is determined according to the distance between the grid point and measurement locations, with the influence of each sensor location governed by radial basis function. The used interpolation function effectively captures the spatial correlations among the sensor data, thus ensuring that the temperature field is reconstructed with high accuracy. Notably, the temperature distribution on the surface typically exhibits strong non‐uniformity,^[^ ^26^ ^]^ which is characterized by sharp local variations and relatively smooth changes elsewhere. The fixed kernel width such as Gaussian kernel is generally utilized in conventional RBF methods to cause poor adaptability and weak capability in handling local variations. Hence, the LARBF method is proposed in this study. By dynamically adjusting the kernel width based on the local data distribution of each interpolation points, it aims to balance local sensitivity and global smoothness. The basic principle is briefly introduced as follows.

The existing measurement data points are set as.

(13) $$ℜ=\left\{\left(x_{i},y_{i},s_{i}\right)\left|i=1,2,\text{…},n\right.\right\}$$

where (*x_i_*, *y_i_*) represents the position coordinate of the *i*‐th sampling point. *s_i_* is the corresponding temperature observation value.

For the grid point to be interpolated (*x_q_*, *y_q_*), the Euclidean distance *r_i_* to all sampling points is calculated as shown in Equation (14).

(14) $$r_{i}=\sqrt{{\left(x_{q}-x_{i}\right)}^{2}+{\left(y_{q}-y_{i}\right)}^{2}}$$

The *k* nearest measurement points around (*x_q_*, *y_q_*) are determined. The average distance can be calculated as follows.

(15) $${\bar{r}}_{k}=\frac{1}{k}\sum_{j=1}^{k}r_{\left(j\right)}$$

where *r*_(_ *_j_*_)_ represents the *j*‐th value in ascending order of distance.

According to Equation (16), the local kernel width can be defined.

(16) $$\varepsilon_{q}=\beta \times {\bar{r}}_{k}$$

where *β* is the kernel width scaling factor with the general value of 0 < *β* < 1. It controls the expansion degree of the local kernel function. The kernel width of the sparse area increases, while that of the dense area decreases to preserve the local data characteristics and global features. Subsequently, Gaussian RBF is employed to define the weights, as shown in Equation (17).

(17) $$\omega_{i}=\exp\left[-{\left(r_{i}/\varepsilon_{q}\right)}^{2}\right]$$

Finally, the predicted values at the interpolation points are obtained through weighted averaging.

(18) $$\hat{s}\left(x_{q},y_{q}\right)=\frac{\sum_{i=1}^{n}\omega_{i}s_{i}}{\sum_{i=1}^{n}\omega_{i}}$$

The above process realizes soft assignment for sampling points, where closer points contribute the greater weights, to conform to the principle of local continuity.

### 3.6 Uncertainty Quantification

To further evaluate the credibility of interpolation results, the local prediction variance and the CI are estimated and provided synchronously during the interpolation process. The weighted sum of squared residuals is used to estimate the local variance, as shown in Equation (19).

(19) $${\hat{\sigma }}^{2}\left(x_{q},y_{q}\right)=\alpha \times \frac{\sum_{i=1}^{n}\omega_{i}{\left[s_{i}-\hat{s}\left(x_{q},y_{q}\right)\right]}^{2}}{\sum_{i=1}^{n}\omega_{i}}$$

where *α* is the variance adjustment coefficient, which is utilized to control the estimation sensitivity

The local standard deviation is.

(20) $$\hat{\sigma }\left(x_{q},y_{q}\right)=\sqrt{{\hat{\sigma }}^{2}\left(x_{q},y_{q}\right)}$$

Based on the normal distribution, the 95% confidence interval is given with the standard value *z* = 1.96, as shown in Equation (21).

(21) $$\begin{array}{c} {\hat{s}}_{upper}\left(x_{q},y_{q}\right)=\hat{s}\left(x_{q},y_{q}\right)+1.96\times \hat{\sigma }\left(x_{q},y_{q}\right)\\ {\hat{s}}_{lower}\left(x_{q},y_{q}\right)=\hat{s}\left(x_{q},y_{q}\right)-1.96\times \hat{\sigma }\left(x_{q},y_{q}\right) \end{array}$$

It reflects the probability that the true value falls within the predicted range at the 95% confidence level, which can provide strong support for subsequent risk assessment and field analysis.

## 4 Conclusion

In this study, the serpentine‐spiral layout DOFS layout method is proposed and demonstrated to achieve in‐situ and full‐range temperature measurement on the surface of large‐capacity pouch battery. Meanwhile, an innovative temperature field reconstruction method with reliable uncertainty quantification was developed. The principal description, algorithm introduction, implementation strategy, results analysis and discussion, and comprehensive comparison with existing techniques are provided in detail. Several crucial conclusions are as follows.

(1) The proposed serpentine‐spiral shaped DOFS layout overcomes the limitation of discrete measurement points, which achieves the leap from point to entire surface in temperature acquisition with strong adaptability. Compared to the dual U‐shaped layout, it provides more comprehensive and meaningful data to support thermal management optimization.

(2) With C‐rates increasing, temperature uniformity deteriorates significantly. Higher current induces greater thermal gradients. Under all tests, elevated temperatures are predominantly observed near the positive tab region. It demonstrates that the potential inadequacy of uniform cooling necessitates localized cooling strategies to regulate the temperature of critical regions.

(3) The adaptive temperature field reconstruction method exhibits satisfactory characterization performance under varying C‐rates and SOCs, with uncertainty quantification further validating its reliability. The maximum STD is controlled within 0.3 °C.

In future work, the effectiveness should be further validated at the battery module and pack levels. Additionally, the implanted deployment and application need to be comprehensively discussed, which aims to facilitate the commercial implementation and meet various demands.

## 5 Experimental Section

### Battery Preparation

In this study, the NCM pouch cell with a nominal capacity of 80.7Ah were utilized, which consisted of the high‐nickel ternary cathode material, LiNi*_x_* Co*_y_* Mn_1‐_ *_x_*_‐_ *_y_* O_2_ (*x*≥0.8), and the graphite anode. The battery used in EVs was manufactured by BYD Co., Ltd. in China with the maximum allowable continuous charging current of 193.7A (2.4C), operating between the cut‐off voltage of 2.8–4.25 V. The specific parameters are depicted in **Table** **4**.

**Table 4:** Battery specifications.

Descriptions		Value
Cell dimensions (mm)		320 × 99 × 12.6
Tab dimensions (mm)		60 × 39 × 0.5
Nominal capacity (Ah)		80.7
Voltage range (V)		2.8–4.25
Max charging current (A)	Continuous	193.7 (2.4C)
	Transitory (10s)	380 (4.7C)
Max discharging current (A)	Continuous	161 (2C)
	Transitory (10s)	580 (7.2C)

**Figure 11:**
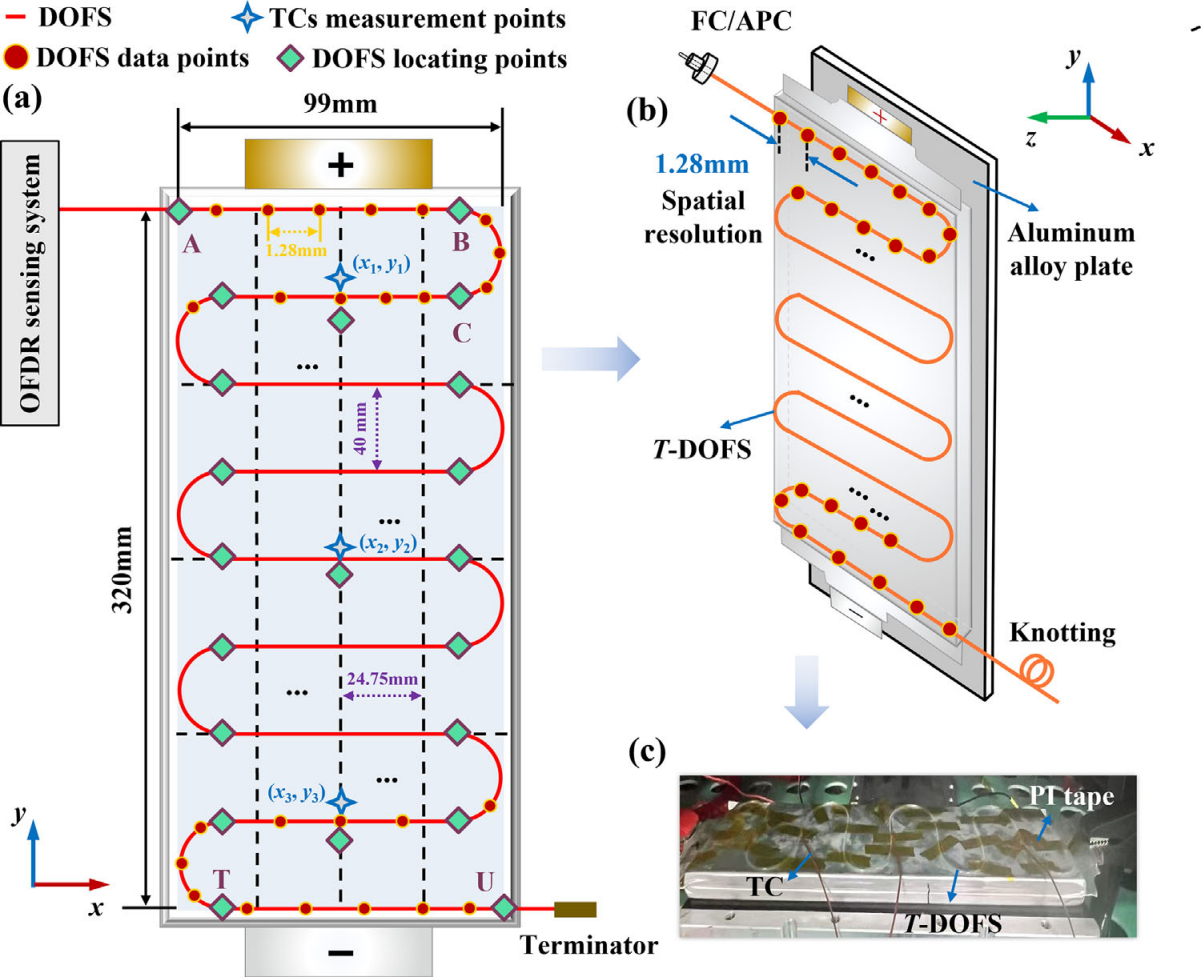
NCM battery with gyrally shaped DOFS (a) Schematic diagram of setup with DOFS and TCs (b) Pouch cell with *T*‐DOFS and other components (c) Battery after preliminary assembling.

*T*‐DOFS was adhered to the surface with epoxy resin or cyanoacrylate glue, which was arranged in multiple continuous S‐shaped patterns along the width direction to form a serpentine layout. The low thermal conductivity of epoxy resin glue may affect temperature measurement results if the adhesive layer is thick. The exothermic curing process also elevated local temperature. Hence, point‐coating was employed as much as possible to preliminarily fix the *T*‐DOFS, which minimized errors and reading fluctuations caused by uncertainties. In this work, to ensure the measurement accuracy and effectiveness of the *T*‑DOFS, a high‑modulus epoxy adhesive (DP‑125, 3 M) and a 30G (0.16 mm) precision stainless steel dispensing needle were employed for bonding. After initially fixing the DOFS using point‐coating method at turning points considered as anchors, a thin layer of epoxy was then evenly applied along the fiber using the needle to secure the remaining segments. Prior to adhesion, the intended fiber placement path was outlined on the cell surface using a marker, which significantly improved placement accuracy and overall bonding effect. Subsequently, PI tape was partially applied to secure the DOFS to prevent any unintended mishandling. Moreover, testing should commence only after complete curing and sufficient resting in the thermal chamber. The battery with the attached DOFS is shown in **Figure** **11**.

In the curved part, the DOFS was bent into a standard semicircle with a diameter of 40 mm, which exceeded the minimum allowable bending radius of 15 mm to ensure the measurement remained unaffected. Finally, three T‐type TCs with the accuracy of ±1 °C are mounted at the center and two additional locations (*x*_1_ = 49.5 mm, *y*_1_ = 280 mm; *x*_2_ = 49.5 mm, *y*_2_ = 160 mm; *x*_3_ = 49.5 mm, *y*_3_ = 40 mm) using PI tape to verify the effectiveness and accuracy of DOFS. The positions of the remaining points were determined through coordinate mapping and calculation as shown in **Table** **5**, which aids the subsequent temperature distributed reconstruction. Only three TCs were employed here to get compromise between connection complexity, cost, and results robust. The above setting mode effectively captured the temperature variations across different regions of surface and accurately reflected the overall state.

**Table 5:** The coordinates of feature points used for temperature reconstruction.

Point	A	B	C	D/(*x*_1_, *y*_1_)	E	F	G
Coordinate	(0, 320)	(79, 320)	(79, 280)	(49.5, 280)	(20, 280)	(20, 240)	(79, 240)
Point	H	I	J	K / (*x*_2_, *y*_2_)	L	M	N
Coordinate	(79, 200)	(20, 200)	(20, 160)	(49.5, 160)	(79, 160)	(79, 120)	(20, 120)
Point	O	P	Q	R / (*x*_3_, *y*_3_)	S	T	U
Coordinate	(20, 80)	(79, 80)	(79, 40)	(49.5, 40)	(20, 40)	(20, 0)	(99, 0)

### Experimental Set‐Up

Twenty one points (A to U) were marked on the battery to define the starting, measuring, and ending positions of the optical fiber signal, as depicted in Figure 11a. To precisely locate and calibrate the OFS measurement points, the cotton swab dipped in small amount of refrigerant was carefully placed at each target location. The low‐temperature property enabled rapid lowering of the temperature in the contact area. The application was conducted with minimal contact area to confine the thermal variation locally. Localized temperature change induced sharp variation in the optical signal, allowing for accurate localization of the corresponding measurement point along the fiber, with the results presented in **Table** **6**.

**Table 6:** Measurement points calibration results.

Points Type	A	B	C	D	¨	R	S	T	U
*P*_locating_ (m)	2.372	2.442	2.505	2.533	¨	3.208	3.234	3.294	3.363
*P*_actual_ (m)	5.372	5.442	5.505	5.533	¨	6.208	6.234	6.294	6.363

Notably, the positional data of the measurement points were determined based on the fiber length settings in the testing software, which follows the equation provided below.

(22) $$P_{\mathrm{actual}}-P_{\mathrm{start}}=P_{\mathrm{locating}}$$

where *P*_actual_ is the start point of the measurement section set by the demodulation software. For example, when the interval is set to 3 to 7 meters, the value of *P*_start_ is regarded as 3. *P*_position_ is the measurement point that was calibrated by refrigerant. *P*_actual_ represents the actual length.

**Figure 12:**
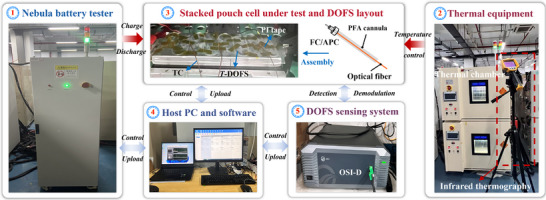
Test platform includes dynamic distributed optical fiber sensing and thermal information.

**Figure** **12** illustrates the schematic diagram of battery dynamic testing platform, which integrates multi‐dimensional thermal‐electrical‐optical information. The platform primarily comprised the Nebula testing equipment, a host computer with testing software, an OSI‐D optical fiber dynamic monitoring instrument, a thermal chamber, and the battery under test. The smart pouch cell affixed with the *T*‐DOFS and TCs was placed on aluminum alloy plate. Subsequently, the assembly was placed in a temperature‐controlled chamber. The Nebula tester was controlled using the testing software and configuration files to perform tests while simultaneously collecting relevant electrical signal data. The OFDR‐based sensing system interrogated the DOFS to detect changes in the interference light, which were then analyzed on the host computer to extract temperature variation information. It is noteworthy that the distributed optical fiber sensing system based on OFDR technology used in this study was independently developed and commercialized by Wuhan Mega‐sense Technology Co., Ltd. (China). The system key performance parameters included sensing length, sampling frequency, spatial resolution, and measurement mode. Detailed parameters are listed in **Table** **7**.

**Table 7:** Performance parameters of distributed optical fiber sensing system based on OFDR.

Descriptions	Value
Sensing length (m)	20
Sampling frequency (Hz)	60
Spatial resolution (mm)	1.28
Repeatability accuracy (°C)	±0.1
Measurement range (°C)	−200 – +800
Equipment power (W)	60
Equipment Size (mm)	W345 × D390 × H165
Measurement mode	High‐performance/ High‐speed/ **High‐precision**

The charge–discharge tests under varying conditions were conducted to verify the proposed method. The batteries are placed in thermal chamber with 25 °C. Before current loading began, the cells rested for 180 min to achieve thermal equilibrium. The CC–CV protocol was executed with various charging rates of 0.5C, 1C, and 1.5C. The current rate was kept below 2C to prevent excessive heat generation and temperature rise. During the CC phase, the cell was charged to upper cut‐off voltage of 4.25 V. The charging mode then was switched to CV until the current decreased to C/20. Subsequently, the cell rests for 180 min. In the discharge phase, the current rate remains constant and aligned with the charging phase. Discharging concluded when the voltage dropped to 2.8 V. Before starting the next charge–discharge test at higher rate, the cell still needs rest for 180 min. The detailed testing procedure are shown in **Table** **8**.

**Table 8:** Battery detailed testing procedure under different currents.

Step No.	Action	C‐rate	Operating parameters	Ending condition
1	Discharge	0.5	Current = 40A	Voltage = 2.8V
2	Rest	–	Time = 3h	–
3	Charge	0.5	Current = 40A Voltage = 4.25V	Current = 4A
4	Rest	–	Time = 3h	–
5	Discharge	0.5	Current = 40A	Voltage = 2.8V
6	Rest	–	Time = 3h	–
7	Charge	1	Current = 80A Voltage = 4.25V	Current = 4A
8	Rest	–	Time = 3h	–
9	Discharge	1	Current = 80A	Voltage = 2.8V
10	Rest	–	Time = 3h	–
11	Charge	1.5	Current = 120A Voltage = 4.25V	Current = 4A
12	Rest	–	Time = 3h	–
13	Discharge	1.5	Current = 120A	Voltage = 2.8V
14	Rest	–	Time = 3h	–

### Overall Framework

**Figure 13:**
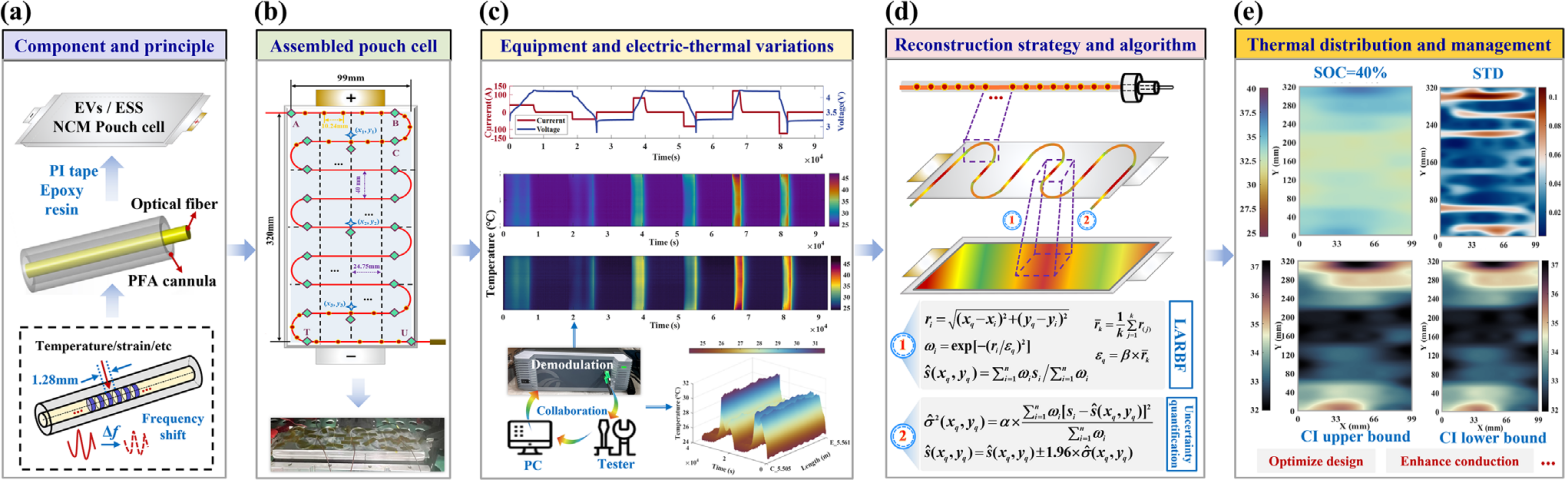
Overall illustration diagram of thermal reconstruction and management for lithium‐ion pouch battery. a) The components and measurement principle. b) Assembled lithium‐ion pouch cell with DOFS. c) Utilized equipment and electric‐thermal variations. d) Temperature reconstruction strategy and algorithm. e) Thermal distribution results and management discussion.

The overall diagram for full‐field temperature reconstruction and management is displayed in **Figure** **13**. The comprehensive system primarily consisted of five parts. a) *T*‐DOFS, which was coated with polyimide and encased in PFA tube wass not affected by strain. The sensor exhibits Rayleigh scattering shifts in response to external temperature changes, which are linearly correlated with temperature. The DOFS was affixed to the surface of large‐format EV/ESS pouch cells using high‐modulus 3 M epoxy resin and precision stainless‐steel dispensing needle (0.16 mm), followed by secondary fixation with PI tape. b) The assembled cell was positioned on an aluminum alloy plate with cushioning layer. Meanwhile, the TCs and two current clamps (positive and negative) were connected to facilitate testing. c) Equipment such as electrical tester, host computer, and optical signal demodulator operate collaboratively to collect multi‐source electro‐thermal‐optical signals. Detailed elaborations were presented for heat generation mechanisms and spatiotemporal evolution of temperature. d) 1D fiber data were mapped onto a 2D entire surface using a reconstruction algorithm, followed by uncertainty quantification to assess the results reliability. e) Further, the full‐field surface temperature distribution and corresponding STD at different SOC were obtained. Comprehensive comparisons highlight the superiority of the proposed method. Thermal management strategies at multiple levels, including battery design optimization and improved heat dissipation, were discussed, offering valuable insights into safe and long‐term system operation.

## Conflict of Interest

The authors declare no conflict of interest.

## Author Contributions

Y.Z. and X.Z. contributed equally to this work. Y.Z. contributed to the conceptualization, data curation, formal analysis, investigation, methodology, software, validation, and writing of the original draft. X.Z. contributed to data curation, formal analysis, investigation, methodology, software, validation, and writing – review and editing. Y.S. contributed to formal analysis, funding acquisition, methodology, resources, supervision, and writing – review and editing. M.Y. contributed to formal analysis, software, validation, and writing – review and editing. X.G. contributed to formal analysis and software. J.L. contributed to software and validation. L.H. contributed to formal analysis.

## Data Availability

The data that support the findings of this study are available from the corresponding author upon reasonable request.;

## References

[1] Q. Si , S. Matsuda , Y. Yamaji , T. Momma , Y. Tateyama , Adv. Sci. 2024, 11, 2402608. 10.1002/advs.202402608 PMC11633329 38934905

[2] Y. Li , L. Wang , Y. Song , W. Wang , C. Lin , X. He , Nano‐Micro Lett. 2024, 16, 154. 10.1007/s40820-024-01374-9 PMC10948733 38499708

[3] J. Huang , S. Boles , J. M. Tarascon , Nat. Sustain. 2022, 5, 194.

[4] A. Kushima , K. P. So , C. Su , P. Bai , N. Kuriyama , T. Maebashi , Y. Fujiwara , M. Z. Bazant , J. Li , Nano Energy 2017, 32, 271.

[5] Y. Lu , X. Wang , S. Mao , D. Wang , D. Sun , Y. Sun , A. Su , C. Zhao , X. Han , K. Li , X. Feng , X. Liu , X. Kong , L. Lu , Z. Chu , Q. Zhang , M. Ouyang , Energy Environ. Sci. 2023, 16, 2448.

[6] X. Liu , L. Zhang , H. Yu , J. Wang , J. Li , K. Yang , Y. Zhao , H. Wang , B. Wu , N. P. Brandon , S. Yang , Adv. Energy Mater. 2022, 12, 2200889.

[7] T. Wang , Y. Zhu , W. Zhao , Y. Gong , Z. Zhang , W. Gao , Y. Shang , Green Energy and Intelligent Transportation 2024, 3, 100171.

[8] L. Su , S. Zhang , A. J. H. McGaughey , B. Reeja‐Jayan , A. Manthiram , Adv. Sci. 2023, 10, 2301737. 10.1002/advs.202301737 PMC10502833 37394730

[9] X. Zhang , J. Hu , J. Li , L. Hou , X. Gu , Y. Zhu , Y. Shang , J. Energy Storage 2024, 102, 114059.

[10] Y. Zhu , Y. Shang , X. Gu , Y. Wang , C. Zhang , IEEE Transactions on Transportation Electrification 2025, 11, 5509.

[11] Y. Li , S. Ding , L. Wang , W. Wang , C. Lin , X. He , eTransportation 2024, 22, 100368.

[12] K. Shen , Y. Ling , X. Meng , X. Lai , Z. Zhu , T. Sun , D. Li , Y. Zheng , H. Wang , C. Xu , X. Feng , J. Energy Storage 2025, 113, 115676.

[13] S. Wang , K. Ou , W. Zhang , Y.‐X. Wang , IEEE Transactions on Industrial Electronics 2024, 72, 570.

[14] Z. Wei , J. Zhao , H. He , G. Ding , H. Cui , L. Liu , J. Power Sources 2021, 489, 229462.

[15] L. H. J. Raijmakers , D. L. Danilov , R.‐A. Eichel , P. H. L. Notten , Appl. Energy 2019, 240, 918.

[16] M. Fichtnerx , K. Edström , E. Ayerbeet , M. Berecibar , A. Bhowmik , I. E. Castelli , S. Clark , R. Dominko , M. Erakca , A. A. Franco , A. Grimaud , B. Horstmann , A. Latz , H. Lorrmann , M. Meeus , R. Narayan , F. Pammer , J. Ruhland , H. Stein , T. Vegge , M. Weil , Adv. Energy Mater. 2022, 12, 2102904.

[17] W. Wang , Y. Zhang , B. Xie , L. Huang , S. Dong , G. Xu , G. Cui , Adv. Energy Mater. 2024, 14, 2304173.

[18] G. Yang , C. Leitão , Y. Li , J. Pinto , X. Jiang , Measurement 2013, 46, 3166.

[19] S. Novais , M. Nascimento , L. Grande , M. Domingues , P. Antunes , N. Alberto , C. Leitão , R. Oliveira , S. Koch , G. Kim , S. Passerini , J. Pinto , Sensors 2016, 16, 1394. 27589749 10.3390/s16091394 PMC5038672

[20] M. Nascimento , M. S. Ferreira , J. L. Pinto , Appl. Therm. Eng. 2019, 149, 1236.

[21] J. Peng , S. Jia , H. Yu , X. Kang , S. Yang , S. Xu , IEEE Sens. J. 2020, 21, 4628.

[22] Y. Li , W. Wang , X.‐G. Yang , F. Zuo , S. Liu , C. Lin , J. Power Sources 2022, 546, 231705.

[23] Y. Li , K. Li , X. Liu , X. Li , L. Zhang , B. Rente , T. Sun , K. T. V. Grattan , Appl. Energy 2022, 325, 119787.

[24] X. Gui , Z. Li , X. Fu , C. Wang , H. Wang , F. Wang , X. Bao , Opt. Lett. 2018, 43, 5259. 30382982 10.1364/OL.43.005259

[25] Y. Yu , E. Vergori , D. Worwood , Y. Tripathy , Y. Guo , A. Somá , D. Greenwood , J. Marco , J. Energy Storage 2021, 39, 102560.

[26] K. Li , Y. Huang , G. Han , W. Lyu , A. He , N. Liu , Y. Yu , Y. Huang , J. Power Sources 2024, 624, 235526.

[27] Y. Yu , T. Vincent , J. Sansom , D. Greenwood , J. Marco , J. Energy Storage 2022, 50, 104291.

[28] C. Chen , S. Gao , L. Chen , X. Bao , Sensors 2020, 20, 6407. 33182580 10.3390/s20226407 PMC7696284

[29] J. Amici , P. Asinari , E. Ayerbe , P. Barboux , P. Bayle‐Guillemaud , R. Jürgen Behm , M. Berecibar , E. Berg , A. Bhowmik , S. Bodoardo , I. E. Castelli , I. Cekic‐Laskovic , R. Christensen , S. Clark , R. Diehm , R. Dominko , M. Fichtner , A. A. Franco , A. Grimaud , N. Guillet , M. Hahlin , S. Hartmann , V. Heiries , K. Hermansson , A. Heuer , S. Jana , L. Jabbour , J. Kallo , A. Latz , H. Lorrmann , Adv. Energy Mater. 2022, 12, 2102785.

[30] C. Zhang , Z. Liu , Z. Lao , Y. Zhou , X. Xiao , T. Feng , C. Chang , R. Wang , G. Zhou , X. Guan , Natl. Sci. Rev. 2025, 12, nwaf088. 40226116 10.1093/nsr/nwaf088 PMC11987594

[31] Y. Yu , E. Vergori , F. Maddar , Y. Guo , D. Greenwood , J. Marco , J. Power Sources 2022, 521, 230957.

[32] X. Ge , Y. Zhang , R. Du , N. Chen , Y. Yu , Z. Li , Y. Huang , Chem. Eng. J. 2024, 488, 150895.

[33] L. Albero Blanquer , F. Marchini , J. R. Seitz , N. Daher , F. Bétermier , J. Huang , C. Gervillié , J.‐M. Tarascon , Nat. Commun. 2022, 13, 1153. 35241673 10.1038/s41467-022-28792-w PMC8894478

[34] J. Guo , K. Zhu , Q. Wu , Y. Rao , P. Liang , J. Chen , Z. Zhang , C. Chen , J. Liu , K. Yan , J. Wang , J. Power Sources 2024, 599, 234231.

[35] J. Fleming , T. Amietszajew , J. Charmet , A. J. Roberts , D. Greenwood , R. Bhagat , J. Energy Storage 2019, 22, 36.

[36] A. Jinasena , L. Spitthoff , M. S. Wahl , J. J. Lamb , P. R. Shearing , A. H. Strømman , O. S. Burheim , Frontiers in Chemical Engineering 2022, 4, 804704.

[37] X. Wang , J. Zhu , X. Wei , D. Wang , W. Xu , Y. Jin , H. Dai , Energy Storage Mater. 2024, 65, 103160.

[38] M. F. H. Rani , Z. M. Razlan , A. B. Shahriman , Z. Ibrahim , W. K. Wan , Int. J. Heat Mass Transfer 2020, 153, 119595.

[39] L. Wang , L. Xie , Y. Song , X. Liu , H. Zhang , X. He , Battery Energy 2023, 2, 20220025.

[40] X. Zhang , Y. Zhu , L. Hou , J. Hu , Y. Shang , eTransportation 2025, 100425.

[41] B. Gulsoy , T. A. Vincent , J. E. H. Sansom , J. Marco , J. Energy Storage 2022, 54, 105260.

[42] H. Dileep , K. K. Jha , P. S. Mahapatra , A. Pattamatta , Appl. Energy 2024, 376, 124301.

[43] Z. Wei , J. Hu , H. He , Y. Yu , J. Marco , IEEE Transactions on Industrial Electronics 2022, 70, 555.

